# A fluorescent multi-domain protein reveals the unfolding mechanism of Hsp70

**DOI:** 10.1038/s41589-022-01162-9

**Published:** 2022-10-20

**Authors:** Satyam Tiwari, Bruno Fauvet, Salvatore Assenza, Paolo De Los Rios, Pierre Goloubinoff

**Affiliations:** 1 Department of Plant Molecular Biology, Faculty of Biology and Medicine, University of Lausanne, Lausanne, Switzerland; 2 Institute of Physics, School of Basic Sciences, École Polytechnique Fédérale de Lausanne - EPFL, Lausanne, Switzerland; 3 Departamento de Física Teórica de la Materia Condensada, Universidad Autónoma de Madrid, Madrid, Spain; 4 Condensed Matter Physics Center (IFIMAC), Universidad Autónoma de Madrid, Madrid, Spain; 5 Instituto Nicolás Cabrera, Universidad Autónoma de Madrid, Madrid, Spain; 6 Institute of Bioengineering, School of Life Sciences, École Polytechnique Fédérale de Lausanne - EPFL, Lausanne, Switzerland; 7 School of Plant Sciences and Food Security, Tel-Aviv University, Tel Aviv, Israel

**Keywords:** Biophysical chemistry, Fluorescence resonance energy transfer, Protein aggregation

## Abstract

Detailed understanding of the mechanism by which Hsp70 chaperones protect cells against protein aggregation is hampered by the lack of a comprehensive characterization of the aggregates, which are typically heterogeneous. Here we designed a reporter chaperone substrate, MLucV, composed of a stress-labile luciferase flanked by stress-resistant fluorescent domains, which upon denaturation formed a discrete population of small aggregates. Combining Förster resonance energy transfer and enzymatic activity measurements provided unprecedented details on the aggregated, unfolded, Hsp70-bound and native MLucV conformations. The Hsp70 mechanism first involved ATP-fueled disaggregation and unfolding of the stable pre-aggregated substrate, which stretched MLucV beyond simply unfolded conformations, followed by native refolding. The ATP-fueled unfolding and refolding action of Hsp70 on MLucV aggregates could accumulate native MLucV species under elevated denaturing temperatures highly adverse to the native state. These results unambiguously exclude binding and preventing of aggregation from the non-equilibrium mechanism by which Hsp70 converts stable aggregates into metastable native proteins.

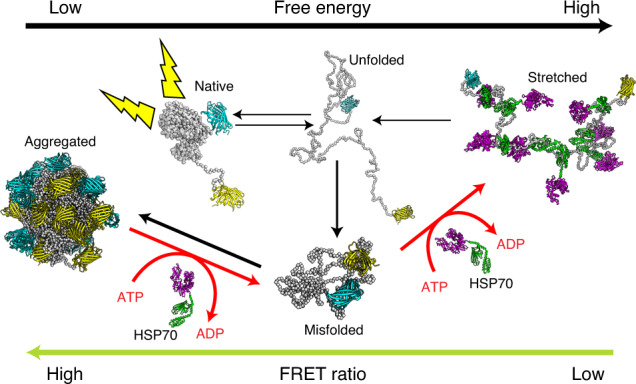

## Abstract (web summary)

By designing a fluorescent protein with a well characterized non-native ensemble, which captures the structural modulation of a substrate by the Hsp70/DnaJ/NEF system, Tiwari et al. resolved the individual steps of the disaggregation unfolding mechanism of Hsp70.

## Main

Nascent polypeptides chains can spontaneously acquire secondary, then near-native tertiary structures^1^. Once released, newly translated proteins can either readily function, or translocate, and/or further assemble with other polypeptides to form functional oligomers^2^. To carry out their unique biological function, native proteins need to remain relatively stable in the crowded environment of cells^3^. Yet, under stress, their partial unfolding may expose hydrophobic residues to the aqueous phase that seek stability by forming misfolded conformations^4–6^. The resulting aggregates come in various sizes, degrees of compactness and solubility, that once the stress is over do not spontaneously revert to the soluble native state.

Misfolding and aggregation are intrinsic consequences of the physics of proteins and their cytotoxic effects particularly affect neurons^7^. In cells, the Hsp70s, Hsp60s, Hsp90s and Hsp100s are distinct families of molecular chaperones that, assisted by a plethora of co-chaperones, use energy from ATP hydrolysis to prevent and actively revert protein aggregation. Along the tree of life, this chaperone network has become progressively more complex concomitantly with an increase in proteome complexity^8^.

Strong evidence has accumulated that Hsp70s, Hsp100s and Hsp60s act as polypeptide-unfolding enzymes specifically targeting stable misfolded and aggregated protein substrates and using ATP to convert them into unstable, unfolded monomeric conformations which, upon release from the chaperone, may then spontaneously fold to the native state^9,10^. Hsp70 functions in close collaboration with Hsp60, Hsp90 and Hsp100, but it can also act independently from them. As such, Hsp70 serves as the central coordinating hub of the chaperone-based ‘protein structure repair’ machineries in prokaryotes and eukaryotes^8,11^.

The activity of Hsp70 strictly depends on a cohort of co-chaperones, called J-domain proteins (JDPs) for their common conserved J-domain that bind ATP-bound Hsp70^12^. JDPs are in general multi-domain^13^. Besides the J-domain, they comprise domains binding other macromolecular complexes, such as protein aggregates, as in the case of DNAJAs and DNAJBs^14–16^, or alternatively folded protein oligomers, such as clathrin cages and heat shock factor 1^17,18^. The targeted Hsp70 can in turn use energy from ATP hydrolysis to pull and/or forcefully import unfolded proteins into organelles, and in general convert the JDP-associated macromolecular complexes into conformationally different ones, with different biological activities^9^.

Upon the concurrent interaction of a J-domain and a bound polypeptide substrate with ATP–Hsp70, the ATPase cycle is accelerated up to a thousand-fold^19,20^, triggering the strong non-equilibrium binding of Hsp70 to the misfolded substrate (ultra-affinity), exploiting the dramatic difference of the binding/unbinding rates between the ATP- and ADP-bound chaperone–substrate complexes^21^. While concomitantly inducing release of JDP, ADP–Hsp70 induces substrate unfolding by clamping and entropic pulling^22,23^. Substrate/product release is then promoted by nucleotide exchange factors^24^.

Crucial to a more precise elucidation of the mechanism of Hsp70, however, is the characterization of its aggregated protein substrates, a task that is complicated by the great heterogeneity of the non-native protein ensemble, comprising aggregates in many sizes, misfolded monomers and transiently unfolded species, each with different degrees of compactness and solubility^25^.

To address this problem, we designed a model multi-domain chaperone reporter protein: MLucV (Supplementary Fig. 1). It is a monomeric, 120-kDa enzyme, comprising a heat-labile and urea-sensitive core made of a mutant active firefly luciferase^6,26^, flanked on either side by urea- and thermo-resistant fluorescent domains^27,28^ (Supplementary Fig. 1). This allowed probing the various MLucV conformations by Förster resonance energy transfer (FRET) and by luciferase enzymatic activity, both in vitro and in cells, finding that the various native, unfolded, chaperone-bound and aggregated states can be unambiguously distinguished. Furthermore, urea and heat pretreatments reproducibly formed small soluble MLucV aggregates composed of ~12 polypeptides. The action of the Hsp70 chaperone system (here bacterial DnaK–DnaJ–GrpE) could then be followed with unprecedented detail, revealing the various steps through which it disassembles stable protein aggregates, without necessitating assistance from co-disaggregase ClpB. Hsp70 was further found to unfold and stretch the substrates beyond their urea-unfolded conformation. Moreover, the ATP-fueled unfolding action of Hsp70 could actively disaggregate stable MLucV aggregates and accumulate native species against equilibrium, under stressful conditions highly adverse to the native state of luciferase.

## Results

### FRET recapitulates distinct MLucV states

**Fig. 1:**
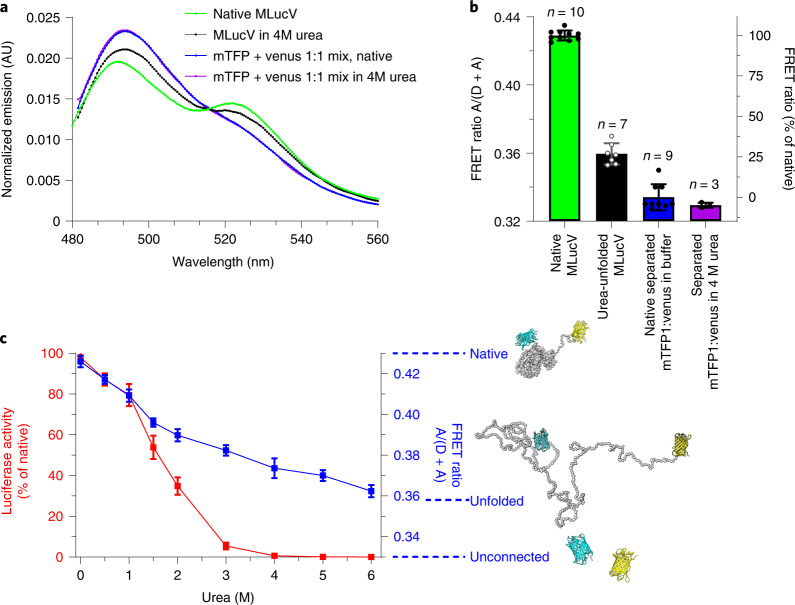
Biophysical characterization of native and non-native MLucV. **a**, FRET spectra (normalized to their respective area) of MLucV (0.4 µM) under native conditions (green) compared to 4 M urea (black) and to disconnected fluorophores (0.4 µM each) under the same conditions (blue and purple, respectively). **b**, Calculation of FRET-PRs from the spectra in **a**. The right *y*-axis shows FRET ratios normalized to that of native MLucV (Methods). Error bars show means ± s.d.; respective sample sizes are shown above the bars. **c**, MLucV urea denaturation curves, monitored by FRET and luciferase activity. MLucV (0.4 μM) was incubated with different urea concentrations (0, 0.5, 1, 1.5, 2, 3, 4, 6 M urea) at 30 °C for 15 min. Luciferase activity and FRET were measured (separately for the same samples) in the presence of each urea concentration. Error bars show mean ± s.d. from *n* = 4 independent experiments. The native and unfolded protein models have been obtained using Molecular Dynamics simulations (Methods). Source data

**Extended Data Fig. 1:**
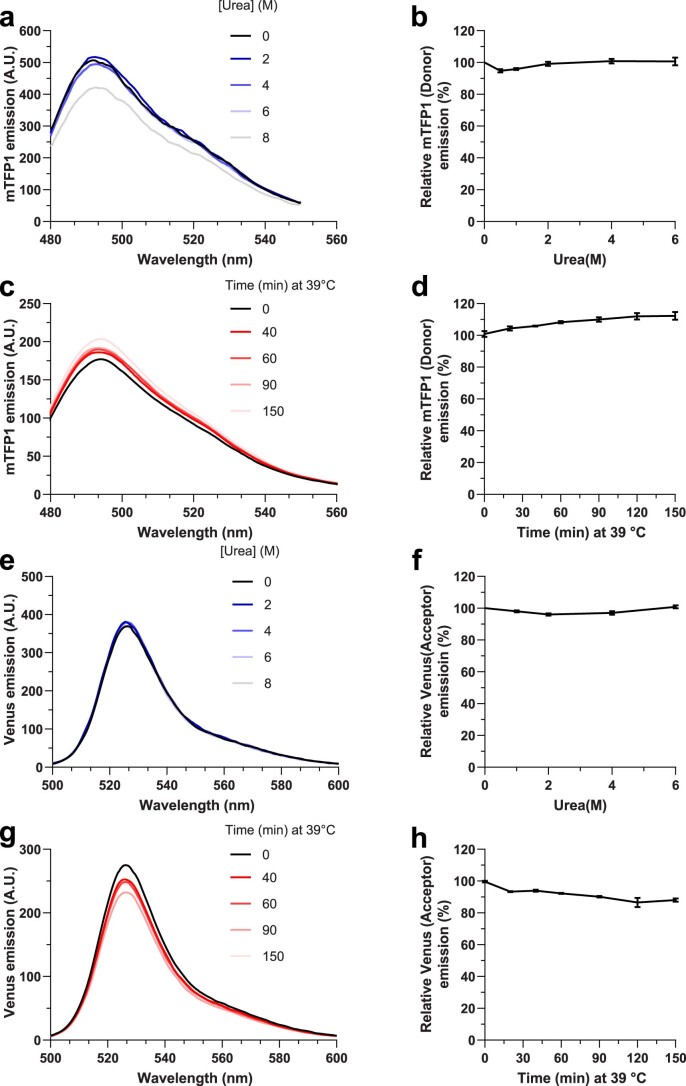
Urea and temperature stability of individual mTFP1 and Venus fluorophores. **a)** The intrinsic fluorescence of 0.4 µM mTFP1 excited at 405 nm was measured at 30°C after 15 min incubation in the presence of up to 8 M Urea. **b)** Relative peak fluorescence (percentage) of mTFP1 normalized to the no urea control, from the data in a). **c)** mTFP1 fluorescence was measured at different time points during incubation at 39 °C, up to 150 min. **d)** Relative mTFP1 fluorescence from the data in c), normalized to the value at t = 0. **e)** to **h)** Same experiments as in a)-d), with Venus instead (excitation wavelength: 480 nm). Source data

The different MLucV states could be characterized by two independent and complementary means: FRET and enzymatic luciferase activity. We first analyzed the FRET spectra of 400 nM native MLucV without urea, and locally unfolded MLucV in the presence of 4 M urea, compared to equimolar separated mTFP1 and Venus proteins (400 nM each), finding they were clearly distinguishable (Fig. 1a). Spectra were converted into FRET proximity ratio values (FRET-PR; Methods), finding that the value for native active MLucV (~0.43) was much higher than the baseline corresponding to unconnected mTFP1 and Venus (0.33) (Fig. 1b). These values and the spectra in Fig. 1a suggest effective donor–acceptor spectral crosstalk, which we thereafter corrected by referring to relative FRET-PRs of 100% and 0% for the FRET value of native MLucV and separated fluorophores, respectively^29^ (Methods). This indicates that when connected by a compact native luciferase core, the two distal fluorophores are on average closer than when unconnected. Denaturation of the luciferase by urea completely abolished its enzymatic activity above 3 M. Concomitantly, its FRET-PR decreased to a value that leveled 27% below that of native MLucV, albeit clearly above that of the unconnected fluorophores, which remained constant between 4–6 M (Fig. 1c). The fluorescence of individual Venus and mTFP1 did not change up to 6 M urea (Extended Data Fig. 1a,b,e,f), indicating that the FRET value of MLucV with 4 M urea was that of a species with an unfolded luciferase flanked by intact native fluorophores.

**Fig. 2:**
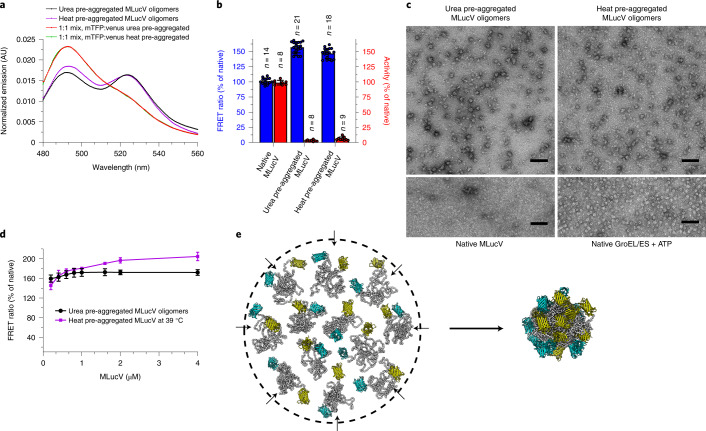
Biophysical characterization of aggregated MLucV. **a**, FRET spectra (normalized to their respective area) of UMLucV (black; 30 µM MLucV were unfolded at 25 °C in 4 M urea for 5 min, then diluted to 0.4 µM and incubated 20 min at 25 °C) and HSMLucV (purple; 0.4 µM incubated 23 min at 38 °C, followed by 20 min at 25 °C). Mixtures of disconnected fluorophores prepared under the same conditions are also shown (red and green spectra, respectively). **b**, Normalized FRET ratios derived from the spectra in **a** and luciferase activity in the corresponding conditions. The left *y*-axis shows FRET ratios (blue bars) normalized to that of native MLucV, and the right *y*-axis shows similarly normalized luciferase activity (red bars). Error bars show means ± s.d.; respective sample sizes are shown above the bars. **c**, The structure of UMLucV and HSMLucV oligomers analyzed by negative stain electron microscopy. Samples were prepared as in **a**. Scale bars, 100 nm. Bottom, native MLucV and native GroEL + GroES taken as controls. All transmission electron microscopy experiments were reproduced at least three times with similar results. **d**, Normalized FRET ratio of UMLucV oligomers (black circles, prepared as in **a**), or HSMLucV oligomers (after 20 min preincubation at 39 °C, then 20 min at 25 °C; magenta circles); from 0.4 up to 4 µM. Error bars show means ± s.d. (*n* = 3). **e**, Molecular Dynamics simulation of the aggregation of 12 misfolded monomers: they were free to diffuse, rotate and rearrange in a confining potential (schematically represented by the circle), which became progressively more confining (arrows) until the 12 monomers arranged in a single aggregate. The confining potential was then turned off and the resulting aggregate allowed to briefly rearrange (Methods). Gray, luciferase core; cyan, mTFP1; yellow, Venus. Source data

**Extended Data Fig. 2:**
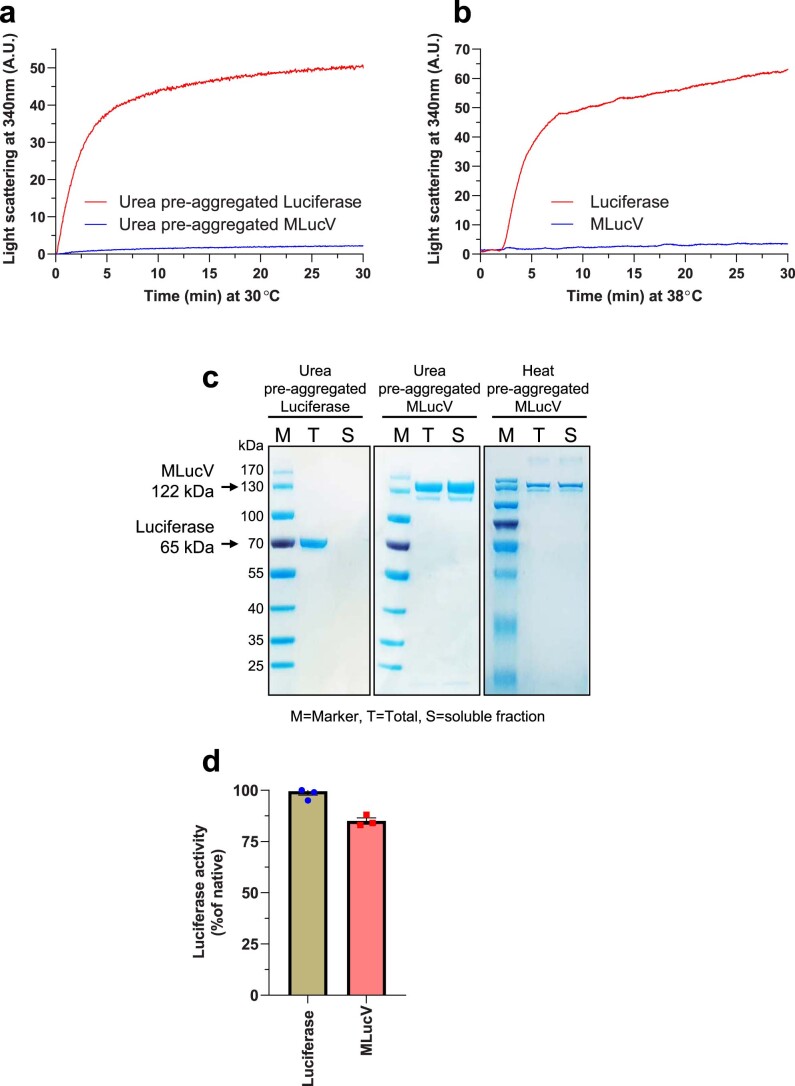
Aggregation propensity of Urea or heat denatured Luciferase and MLucV. a) 20 µM Luciferase or MLucV was preunfolded with 4 M urea at 30°C for 30 min, then diluted to a final concentration of 1 µM in buffer (50 mM HEPES-KOH pH 7.5,150 mM KCl, 10 mM Mgcl2, 2 mM DTT), immediately aggregation was monitored by light scattering at 340 nm at 30°C for 30 min using Perkin Elmer Fluorescence Spectrophotometer. b) Aggregation propensity of heat denatured Luciferase and MLucV. 0.5 µM MLucV or Luciferase was incubated at 38°C and aggregation was monitored for 30 minutes by light scattering at 340 nm wavelength. c) Solubility analysis of urea-preaggregated and heat denatured - MLucV, compared to luciferase alone. ΔLuciferase or MLucV was denatured with 4 M urea at 30°C for 30 min, then diluted to a final concentration of 1 µM in buffer (50 mM HEPES-KOH, pH 7.5 + 150 mM KCl+10 mM Mgcl2 + 2 mM DTT). Samples were incubated at 30°C for 30 min then soluble fraction was separated with high-speed centrifugation (20000 g, 10 min). Equal volumes of Total and supernatant was loaded in 12% SDS-PAGE blue gel. For solubility after heat denaturation, 0.5 µM MLucV was incubated at 38°C for 30 min then soluble fraction was separated with high-speed centrifugation (20000 g, 10 min). Equal volumes of Total and supernatant was loaded in 12% SDS-PAGE gel stained with Coomassie. SDS-PAGE experiments shown are representative from three independent repeats. **d**) Luciferase activity (luminescence) comparison under native conditions between luciferase alone or MLucV (both at 0.5 µM) after 30 min at 25 °C. Error bars show mean ± SD, n = 3 independent experiments. Source data

**Extended Data Fig. 3:**
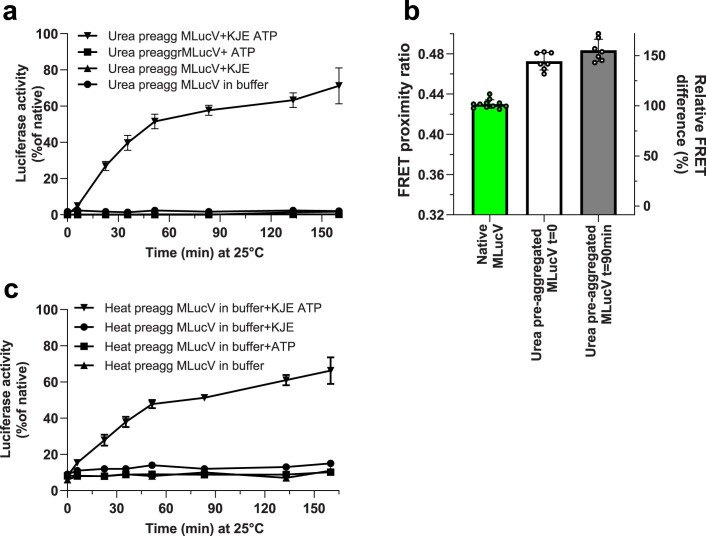
Temporal stability of UMLucV and HSMLucV. **a)** 30 µM MLucV were unfolded at 25°C in 4 M urea for 5 min, then diluted to 0.4 µM in buffer (containing 4 µM BSA with or without 4 mM ATP) and incubated 20 min at 25°C; then samples were supplemented either with water or with KJE (4:1:2 µM final). Luciferase activity was then monitored over time at 25 °C up to 160 min. Error bars show mean ± SD (n = 3). **b)** Calculation (performed as in Fig. 2b) of FRET proximity ratio of urea pre-aggregated MLucV directly after dilution (t = 0) and after 90 min, compared to native MLucV. Error bars show means ± SD (n ≥ 6). **c)** 400 nM MLucV were incubated 35 min at 38°C, followed by 20 min at 25 °C in buffer (containing 4 µM BSA with or without 4 mM ATP). Then, samples were supplemented either with water or with KJE (4:1:2 µM final). Luciferase activity was monitored over time at 25 °C up to 160 min. Error bars show mean ± SD (n = 3). Source data

We next addressed the behavior of aggregated MLucV following different denaturation protocols: urea unfolding, followed by dilution (referred to as UMLucV); or heat denaturation (referred to as HSMLucV). A short preincubation of 30 µM MLucV in 4 M urea at 25 °C, followed by a 75-fold dilution, readily produced stable luciferase species with a FRET spectrum that was distinct from those of native MLucV, without and with 4 M urea and was also distinct from a mixture of disconnected fluorophores under the same treatments (Fig. 2a compared to Fig. 1a). These species were enzymatically inactive and had a FRET-PR consistently higher by ~50% than that of the native species (Fig. 2b compared with Fig. 1b). Note that the flanking fluorophores only marginally decreased the maximal enzymatic activity of the native luciferase core (Extended Data Fig. 2d), suggesting that in UMLucV, mTFP1 and Venus were on average closer than in the native MLucV monomers. Furthermore, no spontaneous refolding of UMLucV was observed, and its FRET remained stable for more than 90 min after initial denaturation and dilution (Extended Data Fig. 3a,b). A similar result was obtained when 400 nM native MLucV was incubated for 23 minutes at 38 °C, followed by 20 min at 25 °C: the corresponding fluorescence spectrum resembled that of UMLucV species (Fig. 2a). Furthermore, over 90% of luciferase was inactive (Fig. 2b) and remained so following the stress (Extended Data Fig. 3c). In a control experiment, the intrinsic fluorescence of individual mTFP1 and Venus was found mostly unaffected by 2 h at 39 °C (Extended Data Fig. 1c,d,g,h), except for a small increase in mTFP1 fluorescence, possibly owing to further fluorophore maturation at 39 °C, while Venus fluorescence slightly diminished (~10%). Although these changes in fluorophore emission affect the measured FRET-PRs of MLucV at 39 °C, the observed conformation-independent small decrease in acceptor emission and increase in donor emission resulted in less than 10% underestimation of the FRET ratio, thus not affecting their interpretation in the case of HSMLucV.

**Extended Data Fig. 4:**
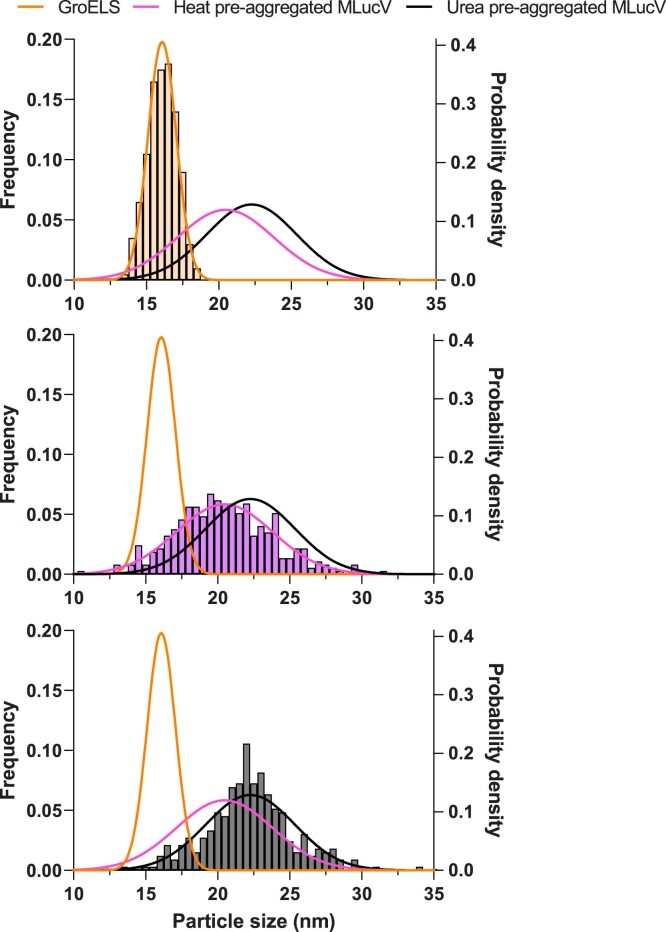
Size distributions of particles observed by negative-staining TEM. Particles sizes were manually measured using ImageJ. Histograms (left y-axes) of particle sizes are shown for GroEL rings (top, n = 200), HSMLucV particles (middle, n = 372), and UMLucV aggregates (bottom, n = 331). For comparisons, all normal distributions fitted to the histograms are shown in each panel (right y-axes). Source data

**Extended Data Fig. 5:**
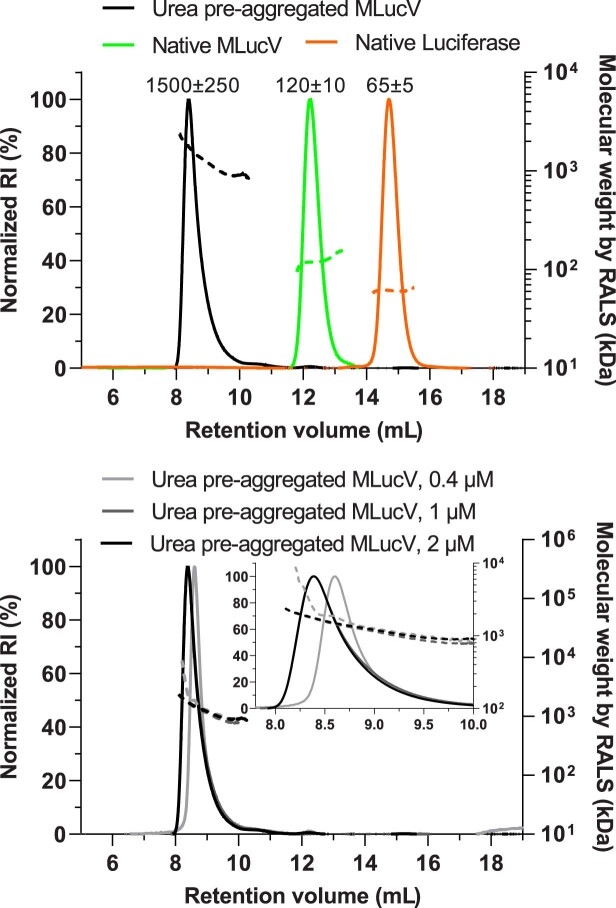
Molecular weight calculations of native and aggregated MLucV by SEC-RALS. In all panels, left y-axes show the relative abundance of eluted proteins, measured by differential refractive index (RI); and right y-axes show the calculated molecular weights (shown as dashed lines) of eluting species. **a)** SEC-RALS analysis of Native MLucV, native Luciferase and UMLucV, showing elution of UMLucV as a ~12mer (black, 1500 ± 250 kDa), native MLucV as a monomer (green, 120 ± 10 kDa), and native Luciferase as monomer (orange, 65 ± 5 kDa). **b)** SEC-RALS analysis of different concentrations of UMLucV. The inset shows the narrow range of elution times and molecular weights. Source data

**Extended Data Fig. 6:**
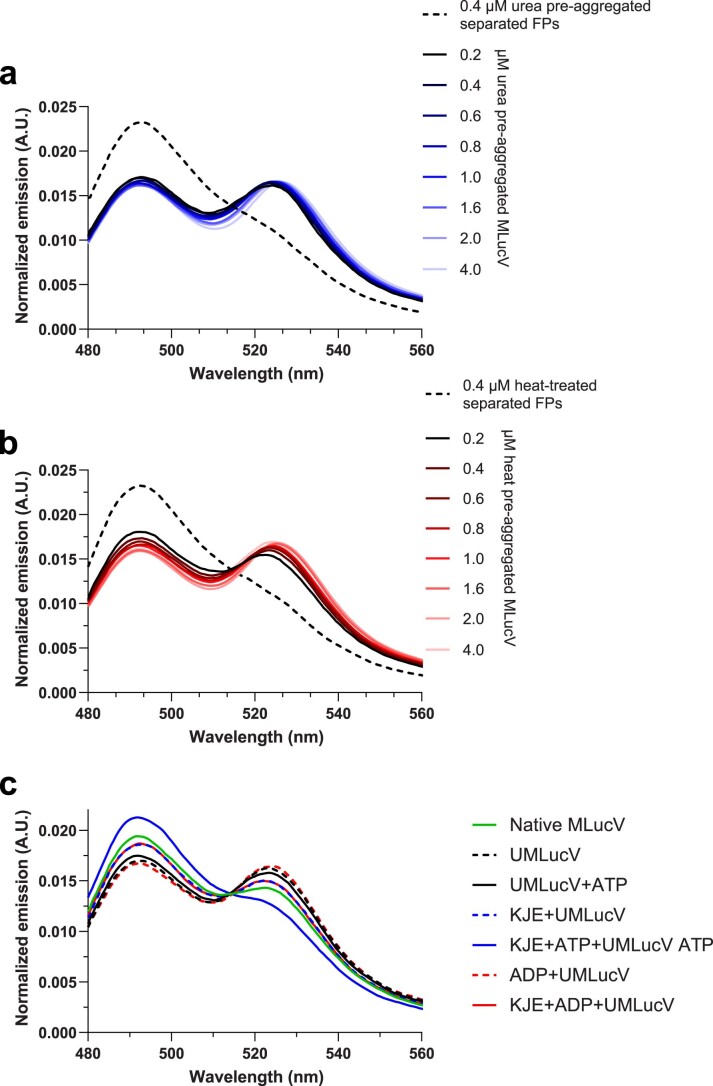
Raw fluorescence spectra corresponding to the data points in Figs. 2d and 3a. **a**) Spectra corresponding to increasing concentrations of UMLucV oligomers (30 µM MLucV were unfolded in 4 M urea at 25°C for 5 min, then diluted to the indicated concentrations and further incubated 20 min at 25°C). **b**) Spectra corresponding to increasing concentrations of HSMLucV. Increasing concentrations of MLucV were incubated at 39°C for 20 min, followed by 20 min at 25°C, and then spectra were measured. Note that due to varying MLucV concentrations, the fluorometer’s detector gain needed to be adjusted to avoid signal saturation, resulting in different overall signal amplitudes. **c**) Fluorescence spectra representative of the FRET ratios shown in Fig. 3a. For clarity, spectra from one repeat are shown. 30 µM MLucV unfolded in 4 M urea at 25°C for 5 min, then diluted to 0.4 µM in refolding buffer in the absence or presence of ATP (4 mM), or KJE (4 µM DnaK, 1 µM DnaJ+2 µM GrpE respectively), or KJE + ATP, ADP (4 mM), or KJE + ADP. FRET spectra were measured after 120 min at 25 °C. Source data

Negative stain electron microscopy showed that both UMLucV and HSMLucV species formed a population of discrete, roughly spherical (although some were slightly elongated) particles (Fig. 2c) with a diameter of about 22.3 ± 3.1 nm and 20.5 ± 3.3 nm, respectively (Extended Data Fig. 4), which were distributed over a slightly wider range of sizes compared to the GroELS particles, used here as a control of ring complexes with an average diameter of 16.1 ± 1 nm. UMLucV particles were slightly larger, with a wider size distribution than the HSMLucV ones. Nevertheless, irrespective of the protocol, the size distribution of the MLucV aggregates remained very narrow compared to what usually occurs to urea-dilution or heat-aggregating proteins (Fig. 2c and Extended Data Fig. 4). Size-exclusion chromatography–right-angle light scattering (SEC–RALS) showed particles with an average mass of 1,500 kDa, suggesting an average of 12 ± 2 misfolded MLucV subunits (Extended Data Fig. 5a). In the case of UMLucV, the FRET-PR of the aggregates as a function of protein concentration reached a plateau of 166% of the native value at 1,500 nM, which was maintained when increasing the concentrations up to 4,000 nM MLucV (Fig. 2d and Extended Data Fig. 6a). Remarkably, 4,000 nM MLucV formed aggregates similar in their FRET to those formed from a 1,500 nM species. This was confirmed by SEC–RALS measurements at up to 2,000 nM UMLucV, showing a similar size profile (Extended Data Fig. 5b). These results are at variance with the general observation that increasing concentrations of misfolding polypeptide protomers strongly increase the size and the compactness of the subsequently forming aggregates^30^. Heat denaturation produced similar results, although the FRET-PRs kept increasing mildly for all increasing concentrations of aggregating species (Fig. 2d and Extended Data Fig. 6b). Attesting for the small size and solubility of these aggregated MLucV oligomers, the light-scattering signals for both UMLucV and HSMLucV were very low and remained so over time, compared to an equimolar luciferase control without flanking fluorescent proteins (FPs) (Extended Data Fig. 2a,b). Moreover, following high-speed centrifugation, the aggregated inactive UMLucV and HSMLucV species remained soluble, unlike aggregates of the Luciferase control (Extended Data Fig. 2c). Unless otherwise specified, throughout the remainder of this work, we used stable UMLucV aggregates produced by pre-denaturing 30 µM MLucV for 5 min in 4 M urea, followed by a 75-fold dilution to a final concentration of 400 nM of aggregated MLucV protomers.

In a typical UMLucV aggregate, the increased fluorophore density, irrespective of their specific arrangement, would translate, as observed, in a higher FRET-PR compared to that of the native monomers, although in such aggregates with multiple, closely spaced donor–acceptor pairs, FRET ratios no longer directly reflect donor–acceptor distances, but just their higher local concentration. We then hypothesized that in a typical UMLucV aggregate, the monomers should tend to arrange according to micellar principles, with their misfolded insoluble luciferases sticking together in a core held together by hydrophobic interactions. Given that the native, soluble and hydrophilic mTFP1 donors and Venus acceptors have no affinity for each other (Fig. 2a, Extended Data Fig. 6a,b and Supplementary Fig. 2a,b), they should be mostly distributed at the surface. Such a configuration for up to ~12 polypeptides would expectedly limit ensuing insertions of additional misfolded luciferases into the hydrophobic core, privileging the formation of similarly sized particles, over further growth of the aggregates.

**Extended Data Fig. 7:**
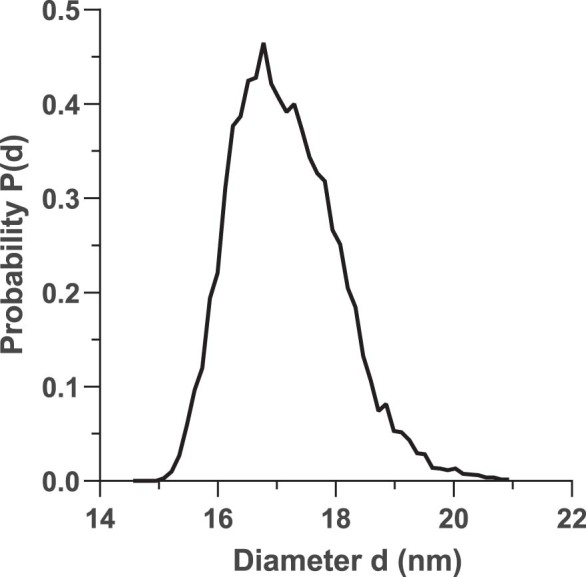
Diameter distribution of misfolded MLucV 12-mers, obtained by MD simulations. Simulations were performed with LAMMPS (see Methods). Fluorophore moieties were excluded in the diameter calculations. Source data

To address this idea, we performed simulations using a coarse-grained description of luciferase, with an inter-residue force-field tuned to describe non-native proteins, while the two soluble FPs were maintained native by suitable structural restraints (Methods). The simulation started with 12 MLucV misfolded monomers, namely polypeptides whose luciferase core was rather compact but not native. We then promoted their aggregation by introducing a confining spherical potential, whose radius shrank in time. No instructions were provided about the possible geometric arrangement of the luciferase and the FPs in the final aggregate. Once the monomers were aggregated, the confining potential was switched off, and the system was allowed to further equilibrate. The simulation produced nearly spherical ~17 nm diameter dodecameric particles (Fig. 2e and Extended Data Fig. 7), with most of the non-native luciferases packed inside and most of the FPs exposed on the surface, positioned closer to each other than in the unfolded or native monomeric states, thus supporting our model.

The limited size of these particles can be understood on simple geometric grounds: packing *n* MLucV monomers results in roughly spherical aggregates with a volume proportional to *n* and a radius proportional to *n*^1/3^. Consequently, the number of FPs (proportional to *n*) increases faster that the surface they can occupy, which grows proportionally to *n*^2/3^, setting a limit to the maximum number of monomers that can take part into a single aggregate particle. The precise value of this upper bound depends on several factors that are not fully characterized, such as the size and cohesive energy of the misfolded luciferase cores, and the fluctuation range of the soluble flanking domains.

**Extended Data Fig. 8:**
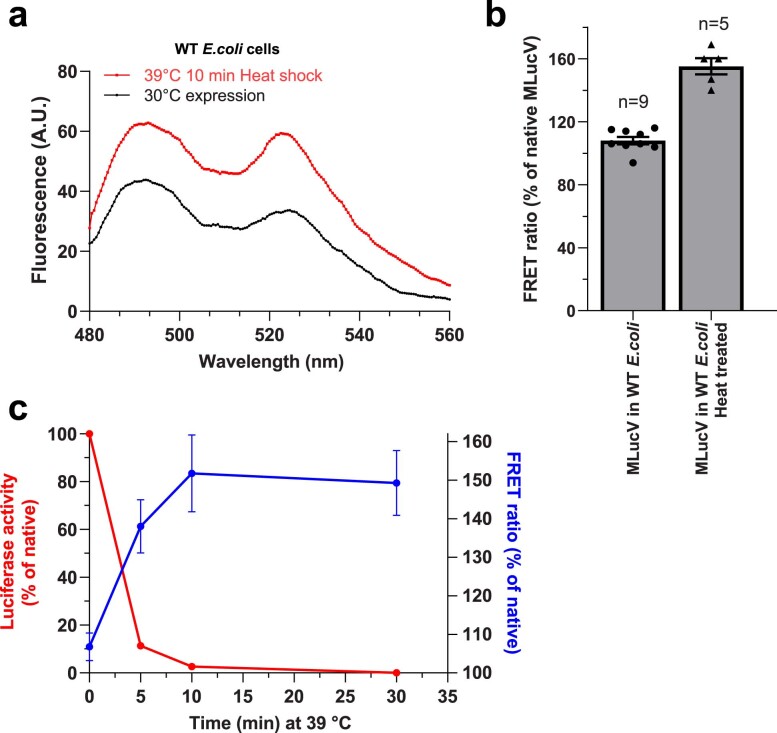
Using MLucV as an in-vivo misfolding sensor. **a**) FRET spectra of MLucV expressed under limited-growth conditions in WT W3110 E. coli; under native conditions at 30 °C (black), or after a 10 min heat shock at 39 °C (red). **b**) Calculation of FRET efficiencies from the spectra in a), normalized to that of native purified MLucV. Error bars show means ± SD; respective sample sizes (independent biological replicates) are shown above the bars. **c**) Time course of MLucV misfolding in WT W3110 E. coli (see Methods) during heat shock at 39 °C, measured by FRET (blue), and enzymatic activity (red). Source data

The in vitro characterization of the different states of MLucV allowed exploring its behavior in vivo. *E. coli* cells expressing low levels of MLucV at 30 °C (to avoid the formation of inclusion bodies) displayed a fluorescence spectrum and a FRET-PR for MLucV that was similar to those found in vitro for native MLucV (Extended Data Fig. 8a,b compared to Fig. 1a,b). After a 10-min incubation of the cells at 39 °C, the in-cell FRET-PR was increased while the luciferase activity from extracts correspondingly decreased (Extended Data Fig. 8c). This indicates that the luciferase lost its native foldedness and the MLucV formed aggregates similar to those found in vitro (Extended Data Fig. 8b compared to Fig. 2b) and suggesting that MLucV can be used as an in vivo reporter for the various stress-dependent states of a protein, in the presence of about 250 mg ml^−1^ of other proteins, including estimated cellular concentrations of 40 μM GroEL, 30 μM DnaK, 20 μM Tig, 5 μM ClpB and 5μM HtpG^31^.

### Probing MLucV states during ATP-fueled unfolding by DnaK

**Fig. 3:**
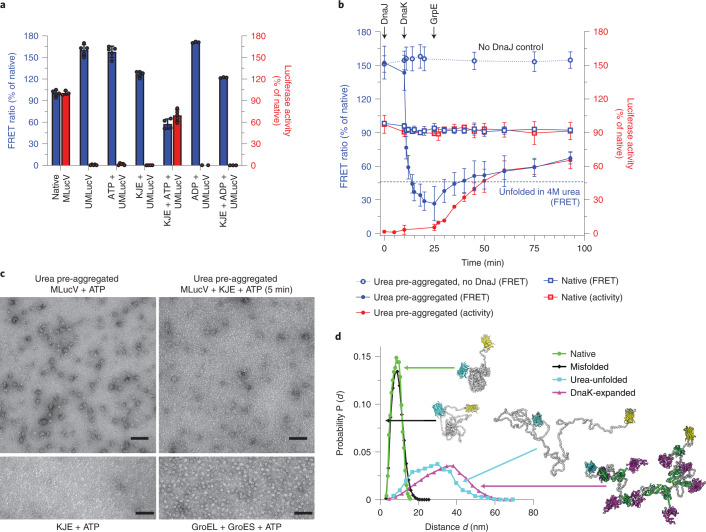
Resolving the individual steps of the KJE mechanism of action. **a**, 30 µM MLucV unfolded in 4 M urea at 25 °C for 5 min, then diluted to 0.4 µM in refolding buffer in the absence or presence of ATP (4 mM), KJE (4 µM DnaK, 1 µM DnaJ and 2 µM GrpE, respectively), KJE + ATP, ADP (4 mM), or KJE + ADP. FRET and luciferase activity was measured after 120 min at 25 °C. Error bars show means ± s.d. (*n* = 3). **b**, Order-of-addition experiment showing DnaJ- and DnaK-mediated unfolding of urea pre-aggregated MLucV oligomers. UMLucV was preformed in the presence of ATP (without chaperones) as in **a**. After, DnaJ, DnaK and GrpE (1, 4 and 2 µM, respectively) were sequentially added at the indicated times (arrows). FRET and luciferase activity were measured at 25 °C. Squares: native MLucV, circles: UMLucV. Error bars show mean ± s.d. from *n* = 3 independent experiments. **c**, Negative-staining electron micrographs of UMLucV oligomers (0.5 µM, prepared as in **b**) before (top left) and after (top right) a 5-min incubation with the KJE chaperone system: DnaK (4 µM), DnaJ (1 µM) and GrpE (2 µM) (top right). Bottom, control samples of KJE + ATP only (left) showing no large particles, and GroEL + GroES (2 µM each, right). Scale bars, 100 nm. All samples were in the presence of 4 mM ATP. TEM experiments were reproduced three times with consistent results. **d**, Probability distribution of the distance between the active centers of mTFP1 and Venus in representative native (green), misfolded (black), urea-unfolded (cyan), and DnaK-expanded (magenta) MLucV monomers, collected from Molecular Dynamics simulations. Source data

The ATP-fueled disaggregation mechanism of Hsp70 chaperones has been predominantly studied using ill-defined pre-aggregated model protein substrates, showing large and poorly reproducible distributions of oligomeric states, with variable degrees of compactness and solubility^32,33^. Here we could clearly and reproducibly identify the native, the unfolded, the chaperone-stretched and a uniquely aggregated states of MLucV, and thus in part resolve the difficulties at investigating the ATP-fueled action of the Hsp70 chaperone on otherwise very heterogeneous populations of misfolded substrates. We thus exploited the properties of MLucV to address the mechanism by which bacterial Hsp70 (DnaK), assisted by its DnaJ and GrpE co-chaperones (the KJE system), can use the energy from ATP hydrolysis to convert stable inactive, discrete, mostly dodecameric MLucV aggregates, into native, functional monomeric conformers. To this aim, 30 µM MLucV was first incubated in 4 M urea, then diluted to 400 nM, without or with ATP, without or with KJE (4:1:2 µM, respectively). Two hours later, both FRET and luciferase activity were measured (Fig. 3a). Representative full FRET spectra are shown in Extended Data Fig. 6c. In the absence of KJE, UMLucV with ATP was enzymatically inactive and reached a FRET-PR of ~160%. The addition of the KJE chaperone system during urea dilution, but without ATP, produced a slightly lower FRET signal than for UMLucV aggregated alone, indicating a very minor ‘holdase’ activity of the chaperones. By contrast, with ATP present, the KJE system promoted, as shown by FRET, a strong decompaction of the aggregates and the accumulation of native monomers, as assessed by enzymatic activity (Fig. 3a).

To address the effect of individual components in the reaction, we applied an order-of-addition protocol. Stable UMLucV aggregates (400 nM protomers), in the presence of 4 mM ATP at 25 °C, were initially supplemented with 1 μM DnaJ (Fig. 3b). After 10 min, only a minor ~5% decrease of the FRET-PR was observed, without any increase of the low basal level of native luciferase activity. This indicates that the expected binding of DnaJ on the surface of the preformed aggregate had little to no effect on their compactness and on the aggregated luciferase core. By contrast, supplementing 10 min after DnaJ addition a tenfold excess of DnaK caused a dramatic and extensive decrease of the FRET-PR, reaching a value lower than that attained by unfolded MLucV in the presence of 4 M urea. This very low FRET-PR strongly suggests that most luciferase polypeptides, which were initially entangled within the core of the particles, became readily bound by up to eight DnaK molecules and were solubilized, as evidenced by the observed distance between FRET pairs, greater than in individual native MLucV protomers. Indicating that for at least 15 min most of the DnaK remained stably bound to the solubilized luciferase and consistent with the extreme expansion of the luciferase core, no luciferase activity was recovered, despite the presence of ATP and DnaJ. After 25 min, 2 μM of GrpE was added, which accelerated ADP exchange into ATP, with the consequent release of the unfolded protein intermediate from DnaK. This led to a prompt recovery of native luciferase activity, up to ~70%, and a corresponding ~60% increase of the FRET-PR towards the native values.

**Extended Data Fig. 9:**
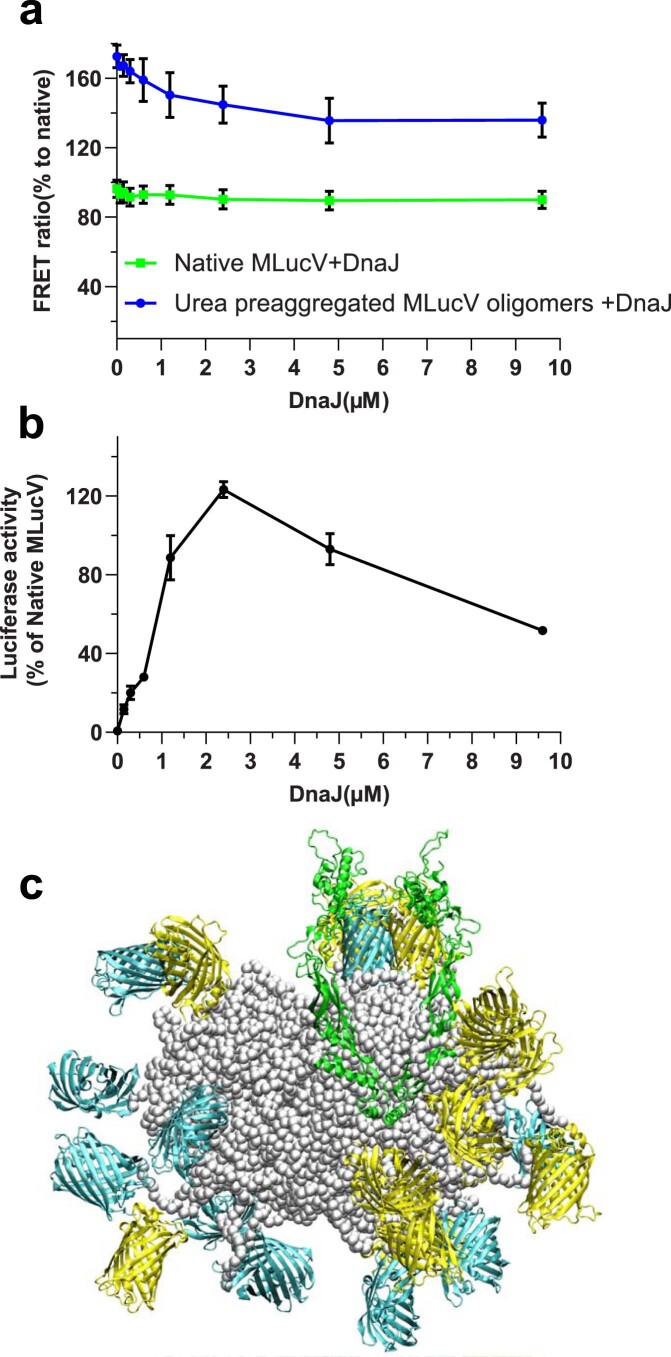
DnaJ binding to urea pre-aggregated MLucV oligomers. **a**) Titration of UMLucV and native MLucV with DnaJ, monitored by FRET. 30 μM MLucV (blue circles) unfolded in 4 M urea at 25°C then diluted to 0.4 μM in refolding buffer in presence of 4 mM ATP, 4 μM BSA, 2 mM DTT and incubated 30 min at 25°C. Increasing amount of DnaJ was added and the reaction was allowed to equilibrate for another 60 min at 25°C. Native MLucV is shown as green squares. The y axis shows FRET ratios normalized to that of native MLucV. Error bars show means ± SD (n = 4). **b**) 4 µM DnaK and 2 µM GrpE was added in samples from a, after 90 min at 25°C, and Luciferase activity was measured. **c**) Representative result from a coarse-grained molecular dynamics simulation, showing DnaJ (green) can access exposed misfolded luciferase segments (gray) on the surface of MLucV oligomers. Source data

Remarkably, when this experiment was repeated without DnaJ (Fig. 3b), addition of DnaK at *t* = 10 min did not change the high FRET signal of the aggregates and did not lead to any luciferase reactivation, despite the presence of ATP and the subsequent addition of GrpE. This clearly shows that although the aggregates are bona fide DnaK substrates, without DnaJ acting as substrate-targeting co-chaperone, DnaK does not bind. Furthermore, the DnaK-mediated refolding of UMLucV showed a strong concentration dependence on DnaJ (Extended Data Fig. 9b); finally, coarse-grained Molecular Dynamics simulations suggest that the fluorophores do not completely sterically hinder DnaJ access to some misfolded luciferase segments exposed at the surface of UMLucV (Extended Data Fig. 9c).

**Extended Data Fig. 10:**
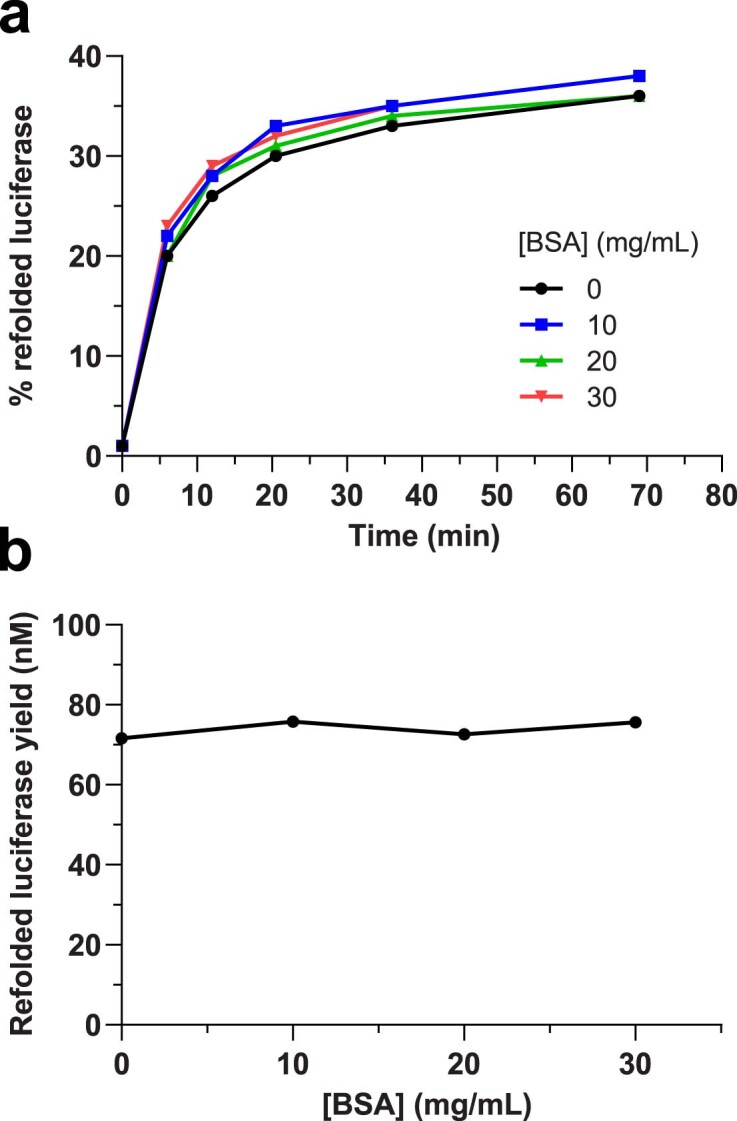
Effect of BSA on KJE-mediated refolding of urea pre-aggregated Luciferase. 200 nM of urea pre-aggregated ΔLuciferase^6^ were incubated at the indicated time points at RT in the presence of 4 µM DnaK, 1 µM DnaJ, 2 µM GrpE and 4 mM ATP and 0, 10 20, 30 mg/mL native BSA. **a)** Time course showing the relative refolding of ΔLucifease for each added BSA concentration. **b)** Refolded ΔLuciferase yield (nM) after 70 min, as a function of added BSA concentration. The presence of up to 2250-fold molar excess of native BSA over the aggregated ΔLuciferase did not affect the yields of the chaperone-mediated unfolding/refolding chaperone reaction, which were all around 72 nM, corresponding to ~36% of natively refolded luciferase. Source data

Adding increasing amounts of only DnaJ to either native or UMLucV resulted in a partial but consistent decrease of the UMLucV FRET-PRs, similarly to previous observations^16^, while it had no effect on native MLucV (Fig. 3b and Extended Data Fig. 9a). This indicates that DnaJ either binds to native MLucV but does not induce a detectable conformational modification or does not bind native MLucV to any relevant extent. Given that a 2,000-fold molar excess of native BSA does not have any effect on the yields of ATP-fueled unfoldase action of the KJE system on pre-aggregated luciferase (Extended Data Fig. 10) and given that in cells a few micromolar DnaJ^31^ can effectively target sub-stoichiometric amounts of non-native proteins in the presence of millimolar of surrounding native proteins, this favors interpreting our data as evidence that DnaJ generally has a minimal affinity for native proteins, in contradistinction to a very high affinity for misfolded proteins.

The high specificity of the chaperone for aggregated substrate polypeptides was further addressed by performing the same order-of-addition experiment with native MLucV (Fig. 3b). In the presence of ATP, addition of DnaJ to native MLucV caused no relevant change in the FRET-PR (set to 100%) and in the measured maximal luciferase activity. The subsequent addition of DnaK at *t* = 10 min did not cause changes in FRET-PR, and the luciferase activity remained high and unchanged; the addition of GrpE at *t* = 25 min had no effect on either the FRET-PR or the luciferase activity. This experiment demonstrates that even in the presence of ATP, neither DnaJ nor DnaK tend to bind native MLucV. Noticeably, the FRET and luciferase activity values of the aggregates treated with DnaJ, then DnaK, then GrpE after 90 min did not reach 100%, (Fig. 3b). Whether this is due to the prolonged DnaK binding to MLucV, keeping it in a stretched conformation despite the presence of GrpE, possibly because of the increased ADP concentration, or just because of an excessively slow reactivation, which would lead to ~100% only on longer times, is left for future investigations.

The fast rate of chaperone-driven disaggregation and unfolding, as estimated from the FRET-PR (~10% min^−1^), was confirmed by negative stain electron microscopy. Within 5 min of KJE addition in the presence of ATP, about 55% of the particles disappeared, (from ~147 particles per µm^2^ before KJE addition to ~67 particles per µm^2^ after; Fig. 3c). Moreover, the MLucV particles that were still present 5 min after KJE addition had distinctly fuzzier edges, suggesting that misfolded MLucV protomers were actively engaged at their surface in DnaK-mediated disaggregation and solubilization.

We then recapitulated the results obtained from the characterization of MLucV in its different states and from the unfolding action of DnaK using Molecular Dynamics simulations. By exploiting the same computational approach used above to investigate the organization of dodecamers (Methods), we considered monomeric MLucV in four different conditions: (i) native; (ii) unfolded (that is, corresponding to the presence of 4 M urea; Methods); (iii) misfolded state; and (iv) with eight ADP–DnaK molecules clamped onto the luciferase core. For each condition, the histogram of the distances between the mTFP1 and Venus active centers was collected (Fig. 3d). The distance probability distribution of the native and misfolded monomers was very similar, suggesting that FRET alone could not tell the difference between the two, although the paired measure of enzymatic activity could relieve this ambiguity. As observed experimentally, the unfolding in 4 M urea resulted in a much broader distance distribution, with an average distance that was larger than in the case of compact native and misfolded monomers, consequently leading to a much lower FRET-PR, as experimentally observed. For an MLucV monomer with eight bound DnaK molecules, the simulated distance distribution was shifted towards even larger values, indicating that the binding of multiple chaperones can further stretch compact misfolded regions in a misfolded polypeptide, and consistent with the observation of a FRET-PR even lower than that of urea-unfolded MLucV.

### Non-equilibrium activity of Hsp70

**Fig. 4:**
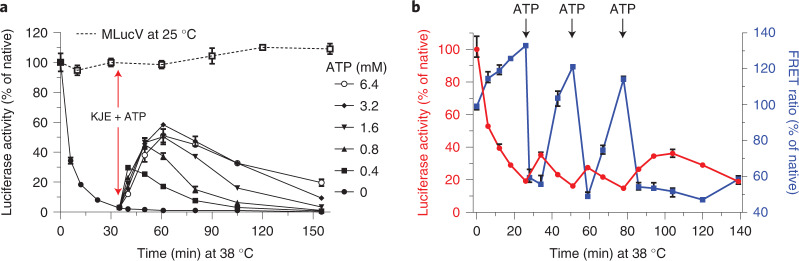
Non-equilibrium action of the KJE system. The KJE system can convert inactive species into native species under conditions that are unfavorable to the native state and this non-equilibrium process depends on ATP. **a**, Native MLucV (0.5 µM) was incubated at 38 °C in buffer with 4 µM BSA for 30 min. At *t* = 35 min, KJE (4, 1, 2 µM, respectively) and increasing amounts of ATP were added (red arrow) at the indicated concentrations and incubated at 38 °C. Luciferase activity is expressed as a percentage of the maximal activity of 0.5 µM native MLucV. Native MLucV at 25 °C (dashed line) is shown as a control. **b**, Native MLucV (0.5 µM) was constantly incubated at 38 °C, in the presence of 4 µM BSA, 4 µM DnaK, 1 µM DnaJ, 2 µM GrpE (KJE) and 2 µM ClpB, first without ATP. Then, 0.8 mM ATP was added at *t* = 26 min and 51 min; and finally 5 mM ATP was added at *t* = 78 min (arrows). Luciferase activity and FRET ratios were measured at the indicated time points and expressed as a percentage of the initial luciferase activity and FRET ratios at *t* = 0. Source data

At 38 °C, the native luciferase core of MLucV was highly unstable, losing its enzymatic activity at an initial rate of ~10% min^−1^ and reaching >95% inactivation in 35 min (Fig. 4a). The subsequent addition of the full KJE chaperone system without ATP did not affect the rate of luciferase denaturation. Yet, when alongside the added chaperones, increasing concentrations of ATP were supplemented, native MLucV rapidly accumulated at 38 °C, at rate of ~2% min^−1^, against its strong tendency to spontaneously denature. The initial refolding rates were about the same for all the tested ATP concentrations, yet with 0.4 mM ATP, less than 40% of MLucV became transiently native and soon became inactive again, owing to the consumption of the ATP. By contrast, with 6.4 mM ATP, a maximal non-equilibrium accumulation of the native population reached nearly 60% of the initial pre-denaturation level and it remained steadily high for more than 1 h, despite the strongly denaturing temperature. Whereas the data in Fig. 3b clearly demonstrated that, without DnaJ, DnaK with ATP is unable to bind and unfold the aggregates, and conversely, that with DnaJ, DnaK with ATP is unable to bind and unfold native MLucV, the results at non-equilibrium elevated temperature demonstrate that the KJE system can specifically target low amounts of heat-misfolding species, as they keep forming, while leaving untouched the conformers that are still native. This also unambiguously demonstrates that the non-equilibrium stabilization of otherwise thermodynamically unstable native MLucV stringently depends on iterative, ATP-fueled chaperone unfolding cycles, which cease once ATP is consumed.

Working under heat-denaturing conditions further demonstrated that the chaperones cannot act by way of merely preventing the aggregation of their ‘client’, just by ‘holding’ the unfolded or the already misfolded monomers^34^. When native MLucV was first incubated at 38 °C in the presence of the KJE system (4μM DnaK, 1μM DnaJ, 2μM GrpE), but without ATP, it lost its luciferase activity at an initial rate of ~10% min^−1^ (as in Fig. 4a) and reached ~20% activity in about 26 min (Fig. 4b), almost as if chaperones were not present. Concomitantly, small aggregates were formed, as evidenced by the FRET signal that increased up to 130%, indicating that in the absence of ATP, the protein-binding ability of a twofold excess of DnaJ and an eightfold excess of DnaK was ineffective at preventing the formation of the MLucV aggregates. Addition, after 26 min, of a limiting amount of ATP (800 μM), caused a sharp decrease of the FRET signal, indicating that disaggregation took place, despite the elevated temperature. Moreover, new native species formed and accumulated during 12 min, reaching a maximal activity level of 35% of native MLucV, against their natural tendency to denature at 38 ^o^C. Yet, indicating that ATP was soon consumed, the luciferase activity dropped again and the FRET signal for aggregation concomitantly increased to a higher level. This suggests that in the presence of ADP, none of the chaperones, although in excess, could passively act as an effective ‘holdase’ for the misfolded MLucV monomers and failed to prevent or change their aggregation pathway. This argues against a possible ‘holdase’ function, which is often ascribed to Hsp70 and Hsp40^34,35^. Addition at *t* = 51 min, of a second dose of 800 μM ATP again accumulated in 8 min up to 27% native MLucV. Once again, the consumption of ATP led to a loss of activity and an increase in the FRET-PR. A third, higher dose of 5 mM ATP added at *t* = 78 min produced longer-lasting native MLucV species, with a FRET signal remaining low (Fig. 4b). Thus, without nucleotides or in the presence of ADP, none of the chaperones added to the solution were able to bind the misfolding species strongly enough to prevent their aggregation, whereas in the presence of ATP, the KJE chaperones could actively unfold non-native, misfolded species from the already formed aggregates, actively prevent their re-aggregation and, upon release, allow them to transiently reach their native state. The prevention of aggregation activity is thus a mere by-product of the ATP-fueled disaggregation, unfolding and refolding-upon-release activity of the KJE system. An obligatory ‘holdase’ step cannot explain the observed ATP-fueled artificial reactivation of the substrate by the chaperones at high temperature.

## Discussion

The activity of proteins is often associated to their ability to switch between several distinct metastable native states, for example, to exploit allosteric control for their function. Moreover, protein unfolding before degradation is necessary to recycle amino acids for synthesis of new proteins^11^. Evolution has thus often compromised between protein stability, necessary to withstand stresses, and conformational flexibility, necessary for function. Therefore, the proteome of mesophilic and thermophilic organisms may still harbor a number of intrinsically thermolabile, or in general stress-sensitive proteins prone to lose their structure and activity and seeking to acquire more stable aggregated inactive conformations. ATPase chaperones represent an astute solution to this dilemma: through their non-equilibrium ATP-fueled unfoldase action, they can transiently increase the native population of metastable proteins without the need to increase the intrinsic thermodynamic stability of their native states, which would otherwise come at the cost of accrued rigidity. Thus, increasingly complex organisms along the tree of life could have evolved an increasing number of very useful chaperone-addicted, metastable proteins^8^. Nonetheless, the elucidation of the precise mechanism through which Hsp70 chaperones operate on substrates that are in a non-native state has thus far been confused by the great heterogeneity of the non-native ensembles.

Here the reporter protein MLucV was used to probe the various states of a chaperone substrate while it is subject to ATP-driven protein disaggregation and unfolding by the bacterial Hsp70 system, under physiological and heat-stress conditions, both in vitro and in vivo. We showed that J-domain co-chaperones target with exquisite specificity Hsp70 molecules onto stress-misfolded and aggregated substrates, and not onto native conformers. We showed that Hsp70s inject energy from ATP hydrolysis into the disassembly of stable aggregates and the forceful unfolding of bound misfolded substrates, thus converting them into stretched intermediates with a higher free energy, which, upon release, may spontaneously collapse into more stable, low-affinity, natively refolded protein products of the unfoldase reaction.

Working under conditions where the native state of the luciferase core is intrinsically unstable (38 °C), further showed that in the absence of ATP, the mere ‘holdase’ activity of the DnaK/DnaJ/GrpE system is ineffective at avoiding the formation of MLucV aggregates. Nonetheless, the binding of DnaJ to non-native polypeptides was necessary to recruit DnaK, which in its absence was unable to disassemble MLucV aggregates even with ATP. Together, our observations suggest that the affinity of DnaJ for non-native monomers is high enough to target DnaK on them but is lower than the affinity of non-native monomers have for each other in the aggregate, thus ruling out DnaJ acting by way of its ‘holdase’ function. Furthermore, no prevention of aggregation was observed by Hsp70 in the ADP-bound state, rebuking the usual view that ATP hydrolysis is merely needed to switch Hsp70 from a low-affinity ATP-bound state to a high-affinity, ADP-bound state. Instead, our results support the view of a truly non-equilibrium enhancement of affinity^21^ (ultra-affinity), exploiting the full ATPase cycle to increase the fraction of DnaK-bound and consequently unfolded polypeptides intermediates, beyond what would be possible in either the ATP- or ADP-bound states. Lastly, our results confirmed our earlier observations that the Hsp70 system is by itself a bona fide, stand-alone disaggregation machinery in the absence of ClpB co-disaggregases^25^. This is in agreement with single-molecule FRET studies of luciferase labeled with small chemical fluorophores performed at very low concentrations to avoid aggregation^36^. Our usage of fluorescent proteins as FRET reporters further allowed MLucV to explore the in vitro action of chaperones on high concentrations of aggregates, and can be used in real time in living cells.

## Methods

### Reagents

The catalog numbers of all commercial reagents used in this study are available in Supplementary Table 1.

#### MLucV (final reporter construct), mTFP1 (FRET pair donor protein) and Venus (FRET pair acceptor protein) plasmid construction

pTriEx-mTFP1(cp175)-Luciferase-Venus(cp173) (called here MLucV) was created by using NEB Gibson assembly (NEBuilder HiFi DNA Assembly_E5520S) taking pTriEx-mTFP1(cp175)-Barnase-Venus(cp173) as template plasmid^27,28^ in which Barnase was replaced by mutant Luciferase^6^.

To construct plasmid with a low leaky MLucV expression in *Escherichia coli*, MLucV was cloned into the pSE380 plasmid. MLucV was PCR amplified from the mTFP1-Luciferase-Venus-pTriEx4 plasmid as DNA template, using primers 5′-AGGAAACAGAATGGGCGGCCACCACCGC-3′ and 5′-CGCCAAAACATTAGATGTTGTGGCGGATCTTGAAGTTGG-3′. The pSE380 vector was PCR amplified using primers 5′-CAACATCTAATGTTTTGGCGGATGAGAG-3′ and 5′-GGCCGCCCATTCTGTTTCCTGTGTGAAATTG-3′ and pSE380-MLucV was created using the NEB Gibson assembly kit (NEBuilder HiFi DNA Assembly_ E5520S). All constructs were confirmed by Sanger sequencing.

GrpE and DnaJ were cloned into pET28-Smt3 expression vector. GrpE and DnaJ were first PCR amplified using *E. coli* genomic DNA then assembled by Gibson assembly (NEBuilder HiFi DNA Assembly_E5520S). The complete maps and sequences of the plasmids described above are available at 10.6084/m9.figshare.20502495.

### Proteins

To begin recombinant production and purification, the MLucV construct was expressed in BL21-CodonPlus (DE3)-RIPL strain (Agilent) using ampicillin and chloramphenicol as the selection antibiotic. Twenty milliliters of an overnight starter culture (grown in LB medium supplemented with 100 μg ml^−1^ ampicillin and 34 μg ml^−^^1^ chloramphenicol at 25 °C) was inoculated in 4,000 ml LB medium plus antibiotics and grown to an optical density at 600 nm (OD_600_) of 0.3 at 25 °C in a shaking incubator. The culture was cooled to 18 °C (for 1 h, no shaking) and expression was induced with 0.2 mM isopropyl β-d-1-thiogalactopyranoside (IPTG) overnight at 18 °C in a shaking incubator. Cells were collected by centrifugation at 5,000*g* for 12 min (pre-cooled rotor-JLA-9.1000 at 4 °C, all subsequent steps were at 4 °C) and washed with chilled phosphate-buffered saline (PBS). Cells were resuspended in 40 ml lysis buffer (50 mM HEPES-KOH pH 7.5, 300 mM NaCl, 5 mM EDTA, 5% glycerol, 5 mM imidazole, 0.5 mM phenylmethylsulfonyl fluoride (PMSF), 2 tablets of complete protease inhibitor cocktail (Roche), 2 mM dithiothreitol (DTT), 40 mg total lysozyme powder (1 mg ml^−1^), and 200 μg DNase). Cells were disrupted by ultrasonication. The lysate was clarified by centrifugation at 20,000*g* for 30 min (JA 25.50 rotor, Beckman centrifuge) and applied directly onto a column of 2 ml pre-equilibrated Ni-NTA beads (cOmplete His-Tag Purification Resin from Merck). After several washes with high salt (500 mM NaCl), 20 mM imidazole and 4 mM ATP, MLucV was eluted with 200 mM imidazole in 25 mM HEPES-KOH pH 7.5, 200 mM KCl, 10 mM MgCl_2_, 2 mM DTT and 5% glycerol. Relatively pure fractions (purity of the fractions was assessed by 12% SDS-PAGE) were pooled, concentrated to ~5 mg ml^−1^ (using 50 kDa Amicon Ultra, Millipore), spun 10,000 r.p.m. for 10 min, and loaded in Superdex-200 increase gel filtration column (GE Healthcare), with final buffer (25 mM HEPES-KOH pH 7.5, 200 mM KCl, 10 mM MgCl_2_, 2 mM DTT). Pure fractions were collected and stored at −80 °C.

DnaK of *E. coli* was expressed and purified as described previously^37^. Purified DnaK was stored in 25 mM HEPES-KOH, 100 mM KCl, 10 mM MgCl_2_ pH 7.4, at −80 °C.

GrpE was expressed and purified from *E. coli* BL21 (DE3) cells harboring the pET28-Smt3-GrpE plasmid. In brief, cells were grown in LB medium with kanamycin at 37 °C to OD_600_ ~0.4–0.5. Protein expression was induced by the addition of 0.5 mM IPTG for 3 h. Cells were harvested, washed with chilled PBS and resuspended in buffer A (20 mM Tris-HCl pH 7.5, 200 mM NaCl, 5% glycerol, 2 mM DTT, 20 mM MgCl_2_) containing 5 mM imidazole, 1 mg ml^−1^ Lysozyme, 1 mM PMSF for 1 h. Cells were lysed by sonication. After high-speed centrifugation (20,000*g*, 30 min, 4 °C), the supernatant was loaded onto a gravity flow-based Ni-NTA metal affinity column (2 ml beads, cOmplete His-Tag Purification Resin from Merck), equilibrated and washed with ten column volumes of buffer A containing 5 mM imidazole. After several washes with high salt, buffer A + 200 mM NaCl, 20 mM Imidazole and 4 mM ATP, N-terminal His_10_-SUMO (small ubiquitin-related modifier) Smt3 tag was cleaved with Ulp1 protease (2 mg ml^−1^, 300 µl), added to beads with buffer (20 mM Tris-HCl pH 7.5, 150 mM KCl, 10 mM MgCl_2_, 5% glycerol, 2 mM DTT). Digestion of His_10_-Smt3 was performed on the Ni-NTA resin by, His_6_-Ulp1 protease. Because of dual His tags, His_6_-Ulp1 and His_10_-SUMO display a high affinity for Ni-NTA resin and remain bound to it during cleavage reaction. After overnight digestion at 4 °C, the unbound fraction (which contains only the native GrpE protein) is collected. GrpE was further purified by concentrating to ~3 mg ml^−1^ and applying to a size-exclusion column (Superdex-200 increase, 10/30, GE Healthcare) equilibrated in buffer A containing 4 mM ATP. Pure fractions were pooled, concentrated by ultrafiltration using Amicon Ultra MWCO 10000 (Millipore), aliquoted and stored at −80 °C. DnaJ was purified in a similar way like GrpE. All protein concentrations were determined spectrophotometrically at 562 nm using BCA Protein Assay Kit− Reducing Agent Compatible (cat. no. 23250).

#### Control plasmids and proteins

Separated donor (mTFP1) and acceptor (Venus) fluorophores were prepared by PCR amplification from the pTriEx-MLucV plasmid using the Q5 Site-Directed Mutagenesis Kit (New England BioLabs). The proteins were expressed and purified in a similar way to MLucV. The plasmid pT7-lucDelta-His, carrying the luciferase gene of *Photinus pyralis* with the additional His6-coding sequence, was a gift from A. S. Spirin^38^. His-tagged Luciferase was purified as described previously^6^.

### FRET measurements and FRET proximity ratio calculation

All ensemble relative FRET ratios were calculated from maximum fluorescence emission intensities of donor (*E*_D_) and acceptor (*E*_A_) fluorophore by exciting donor only at 405 nm wavelength^27,28^. Fluorescence emission spectra analysis of MLucV reporter was performed on Perkin Elmer LS55 fluorometer. Emission spectra were recorded from 480 to 580 nm wavelength with excitation slit 5 nm and emission slit 10 nm. Direct excitation of the acceptor is minimized by exciting the donor at 405 nm. Spectra were background-subtracted with spectra of buffer only or non-transformed cells in case of in vivo measurements, samples acquired in the same conditions. In all cases where it was appropriate to do so, baseline-corrected spectra were further normalized with respect to their respective area. Normalized spectra are indicated in the corresponding figure legends. The relative FRET ratios were calculated using the following equation:

$$\mathrm{FRET}_{\rm{ensemble}} = \frac{{E_{\mathrm{acceptor}}}}{{E_{\mathrm{donor}} + E_{\mathrm{acceptor}}}}$$

Normalized FRET ratios relative to that of native MLucV were calculated as follows^29^:

$${\mathrm{FRET}}_{{\rm{norm}}} = \frac{{\mathrm{FRET}}_{{\rm{ensemble}}} - {\rm{FRET}}_{{\rm{separated}}}}{{\mathrm{FRET}}_{{\rm{native}}} - {\mathrm{FRET}}_{{\rm{separated}}}}$$

where FRET_ensemble_ is the measured ensemble FRET, FRET_separated_ is the calculated ensemble FRET measured in a solution of separated mTFP1 and Venus (~0.335), and FRET_native_ is the measured ensemble FRET of native MLucV (~0.425). Unless otherwise specified, all ensemble FRET measurements were performed at 400 nM of MLucV. Temperature was maintained at 25 °C unless otherwise specified. All experiments were performed in LRB (20 mM HEPES-KOH pH 7.4, 150 mM KCl, 10 mM MgCl_2_) refolding buffer containing 4 mM ATP, 2 mM DTT, unless otherwise specified. Bovine serum albumin (BSA) (4 µM) was used in assays with chaperones to avoid MLucV species sticking to vessel, it has no effect on fate of the formed aggregates, nor have an effect on the activity of the chaperones. All experiments were repeated at least three times.

#### Luciferase activity assay

In the presence of oxygen, luciferase catalyzes the conversion of d-luciferin and ATP into oxyluciferin, CO_2_, AMP, pyrophosphate and visible light. Generated photons were counted with a Victor Light 1420 Luminescence Counter from Perkin Elmer in a 96-well microtiter plate (Thermo Scientific White Polystyrene Plate, cat. no. 136101). For measurements, d-luciferin (25 µM prepared in 50 mM Tris acetate pH 7.5, 50 mM KCl, 15 mM MgCl_2_, 200 µM Co-enzyme A hydrate, 4 mM ATP) was added immediately (75 µl) before luminescence readings using an automated pump in wells having 3 µl sample with 45 µl dilution buffer (50 mM Tris pH 7.5, 50 mM KCl, 10 mM MgCl_2_, 5% Glycerol, 1% w/v BSA). Immediately after adding the d-luciferin solution to a well, the plate was vigorously shaken for 1 s, and luminescence was read for 10 s.

### FRET and Luciferase activity analysis of live *E. coli* cells

Ten milliliters of MLucV-expressing *E. coli* cells (strain W3110) were grown at 30 °C in LB broth supplemented with antibiotics to OD_600_ ~0.4, 1 mM IPTG was then added to induce the expression but only for 2 h. Cells were pelleted and washed twice with PBS + 100 mM glucose pH 7.4 and resuspended in 10 ml of the same buffer. Equal number of cells in each sample was achieved by cell number normalization to OD_600_ = 1 by dilution. One hundred microliters of the cell suspension was added in a cuvette with 100 μl buffer, mixing was performed by pipetting, and the fluorescence was recorded immediately at 25 °C unless otherwise specified.

To measure luciferase activity, the same sample as used for FRET was sonicated for 10 s with 2 μg μl^−1^ lysozyme, immediately after lysis, 5 µl whole cells extract was taken to measure luciferase activity as described earlier in a 96-well plate. Four independent experiments were carried out with two technical replicates for each sample.

#### SEC–RALS analysis of native MLucV, UMLucV oligomers and luciferase

Each sample (native MLucV, UMLucV or luciferase, 1 µM all samples) separately was loaded (300 µl) in a Superdex-200 increase column (GE Healthcare) attached to a Malvern Panalytical Omnisec resolve-reveal system. Using refractive index and right-angle light-scattering detectors, absolute molecular masses were calculated with OMNISEC-v11.10 software (Malvern Pananalytical) with the *dn*/*dc* value set to 0.185 ml g^−1^, following calibration using the BSA monomer elution peak.

#### Light scattering

To monitor aggregation propensity of urea-denatured luciferase and MLucV, 30 µM luciferase or MLucV was incubated with 4 M urea at 25 °C for 10 min, then diluted to a final concentration of 1 µM in buffer A (50 mM HEPES-KOH pH 7.5, 150 mM KCl, 10 mM MgCl_2_, 2 mM DTT), immediately aggregation was monitored by light scattering at 340 nm at 30 °C for 30 min using Perkin Elmer Fluorescence Spectrophotometer. To monitor aggregation propensity of heat-denatured luciferase and MLucV, MLucV or luciferase (0.5 µM) was kept at 38 °C in Buffer A with 4 µM BSA and aggregation was monitored by light scattering at 340 nm for 30 min using Perkin Elmer Fluorescence Spectrophotometer.

#### Solubility test of urea pre-aggregated Luciferase and MLucV

Thirty micromolar luciferase or MLucV was denatured with 4 M urea at 25 °C for 10 min, then diluted to a final concentration of 1 µM in buffer (50 mM HEPES-KOH pH 7.5, 150 mM KCl, 10 mM MgCl_2_, 2 mM DTT). Samples were incubated at 25 °C for 30 min then soluble fraction was separated using high-speed centrifugation (20,000*g*, 10 min). Equal volumes of total and supernatant were loaded on 12% SDS-PAGE blue gel. For solubility of heat pre-aggregated MLucV, 0.5 µM MLucV was incubated at 38 °C for 30 min then soluble fraction was separated with high-speed centrifugation (20,000*g*, 10 min). Equal volumes of total and supernatant were loaded in 12% SDS-PAGE blue gel (Extended Data Fig. 2c).

#### Chaperone assays

To prepare urea pre-aggregated MLucV species (UMLucV) 30 µM MLucV were unfolded in 4 M urea for 5 min at 25 °C, then diluted to 0.4 µM and incubated again for 20 min at 25 °C. For heat pre-aggregated species 0.4 µM MLucV was incubated 23 to 25 min at 38 °C, followed by 20 min at 25 °C. Refolding assays were performed in LRB (20 mM HEPES-KOH pH 7.4, 150 mM KCl, 10 mM MgCl_2_) buffer containing 4 mM ATP, 2 mM DTT and 4 µM BSA, unless otherwise specified. DnaK, DnaJ, GrpE (4, 1, 2 µM, respectively) were added to the samples, and then FRET ratios and luciferase activity were monitored over time at 25 °C for up to 160 min unless otherwise specified.

Note, in all our chaperone assays, 0.4–0.5 μM MLucV (final concentration) was used. Urea- or heat-aggregated MLucV species were freshly prepared each time (never frozen). In all chaperone experiments, except in Fig. 3a (where chaperones were purposefully already present in the buffer used to dilute the urea), MLucV oligomers were prepared first, then chaperones were later added.

### Negative stain TEM Electron microscopy

#### Sample preparation for UMLucV

Thirty micromolar MLucV were denatured with 4 M urea at 25 °C for 5 min, then diluted to a final concentration of 0.5 µM in buffer (20 mM HEPES-KOH pH 7.5, 150 mM KCl, 10 mM MgCl_2_, 2 mM DTT, 4 mM ATP); followed by incubation at 25 °C for 30 min.

#### Sample preparation for HSMLucV

MLucV (0.5 µM) was incubated at 38 °C for 23 min in same buffer as for UMLucV followed by 20 min at 25 °C. Native control samples (MLucV and GroEL and GroES) were loaded directly on grid with the same concentration as urea- or heat-denatured species.

For all above mentioned samples, 3 µl sample (5× diluted) was placed to freshly glow-discharged carbon EM grid (400 mesh copper, Electron Microscopy Sciences). After incubating for 2-min, excess protein was removed by Milli-Q water wash, then grid was immediately placed on 100-µl droplet of 1% (w/v) uranyl acetate solution. After 1 min, excess uranyl acetate was removed from the grid by touching the edge with filter paper and grids were left to air-dry for 20 min. Images were acquired on a Philips CM100 Biotwin (80 kV) transmission electron microscope. Micrographs were acquired at a magnification of 37,000× on an TVIPS F416 camera (4k × 4k). A minimum of ten fields were screened per sample, to collect representative images, with three different repeats. The apparent particle sizes were quantified manually using the Fiji distribution of ImageJ (v.1.53k)^39^. At least 200 particles were measured per condition; because particles were often elongated, two length measurements along perpendicular axes were taken, and the apparent size was determined as the average of the two. Normal distributions were then fitted to size histograms using MatLab R2019b and figures were generated with GraphPad Prism v.9.4.

### Molecular Dynamics simulations

We performed coarse-grained simulations following an approach already used previously^40^. In short, all the amino acids were modeled as beads centered in the Cα atom. Native luciferase, mTFP1 and Venus were modeled as rigid bodies, and their native conformations obtained from the following Protein Data Bank (PDB) structures: 1BA3 (ref. ^41^); 2HQK (ref. ^42^) and 1MYW (ref. ^43^) respectively. In native conditions, linkers were modeled as disordered fragments by means of a model introduced previously^44^ where we considered the case corresponding to the Monera hydrophobicity scale^45^. The extra amino acids at the C terminus of the molecule used in our experiments were modeled as a disordered fragment. Specifically, the extra sequence was GGKSKLSYEQDGLHAGSPAALERAAA (Supplementary Fig. 1). Note that in PDB entry 1BA3, the fragment GGKSKL is present in the fasta sequence, but absent in the pdb file; hence, it was considered here as part of the extra sequence. Under denaturing conditions, following the indications from experiments we assumed that mTFP1 and Venus kept their native structure, while luciferase was considered to be completely unfolded. In this case, the linkers and the unfolded luciferase were modeled by setting to zero the hydrophobicity of all the residues.

DnaK molecules in their ATP form were modeled by considering two independent rigid bodies for the nucleotide-binding domain and the substrate-binding domain (SBD), while the flexible linker was modeled as a disordered fragment. The rigid body of the SBD included also a bound heptapeptide. The reference structure was obtained by combining PDB accession 2KHO (ref. ^46^), which provides the full structure of the ADP-bound DnaK, and PDB accession 1DKX (ref. ^47^), which allows the modeling of the SBD bound to a heptapeptide. The combined structure was obtained by aligning the SBDs of both structures, as in our previous work^40,48^. The binding sites on luciferase were identified by means of a well-established algorithm^49^, resulting in 13 non-overlapping binding sites.

Simulations were performed with LAMMPS^50^, with a Langevin thermostat set at temperature *T* = 293 K and with damping parameter 16 ns^−1^. The time step was set to 1 fs. To accelerate the convergence of simulations, each residue within a disordered fragment was assigned a mass equal to 1 Da, while residues belonging to rigid bodies were given a mass equal to 0.01 Da. Simulations were run for 2 × 10^7^ time steps. To ensure full equilibration, the first 10^6^ steps were discarded from the analysis. Five independent simulations were performed for each case.

### Reporting summary

Further information on research design is available in the Nature Research Reporting Summary linked to this article.

## Online content

Any methods, additional references, Nature Research reporting summaries, source data, extended data, supplementary information, acknowledgements, peer review information; details of author contributions and competing interests; and statements of data and code availability are available at 10.1038/s41589-022-01162-9.

## Supplementary information

Supplementary Information Supplementary Figs 1 and 2, and Supplementary Table 1.

Reporting Summary

Supplementary Table 1 Catalog numbers of commercial reagents.

## Data Availability

All data shown in the present study are available within the main and supplementary figures and provided as source data (Excel files) together with the manuscript. PDB accessions of the models used to run molecular dynamics simulations are provided within the manuscript. Maps and sequences of the plasmids generated in this study are available at 10.6084/m9.figshare.20502495. All generated plasmids and reagents are available upon reasonable request to the corresponding authors. Source data are provided with this paper.

## Source data

Source Data Fig. 1 Statistical source data.
Source Data Fig. 2 Statistical source data.
Source Data Fig. 3 Statistical source data.
Source Data Fig. 4 Statistical source data.
Source Data Extended Data Fig. 1 Statistical source data.
Source Data Extended Data Fig. 2 Statistical source data.
Source Data Extended Data Fig. 2 Unprocessed gel scans.
Source Data Extended Data Fig. 3 Statistical source data.
Source Data Extended Data Fig. 4 Statistical source data.
Source Data Extended Data Fig. 5 Statistical source data.
Source Data Extended Data Fig. 6 Statistical source data.
Source Data Extended Data Fig. 7 Statistical source data.
Source Data Extended Data Fig. 8 Statistical source data.
Source Data Extended Data Fig. 9 Statistical source data.
Source Data Extended Data Fig. 10 Statistical source data.

## Code availability

Code used to run and analyze Molecular Dynamics simulations, including sample LAMMPS inputs (including computation of distances on the fly) and the LAMMPS patch implementing the coarse-grained potential are openly available on GitHub at https://github.com/saassenza/Hsp70Unfoldase.
