# Author Correction: A fluorescent multi-domain protein reveals the unfolding mechanism of Hsp70

**DOI:** 10.1038/s41589-023-01296-4

**Published:** 2023-02-27

**Authors:** Satyam Tiwari, Bruno Fauvet, Salvatore Assenza, Paolo De Los Rios, Pierre Goloubinoff

**Affiliations:** 1 Department of Plant Molecular Biology, Faculty of Biology and Medicine, University of Lausanne, Lausanne, Switzerland; 2 Institute of Physics, School of Basic Sciences, École Polytechnique Fédérale de Lausanne - EPFL, Lausanne, Switzerland; 3 Departamento de Física Teórica de la Materia Condensada, Universidad Autónoma de Madrid, Madrid, Spain; 4 Condensed Matter Physics Center (IFIMAC), Universidad Autónoma de Madrid, Madrid, Spain; 5 Instituto Nicolás Cabrera, Universidad Autónoma de Madrid, Madrid, Spain; 6 Institute of Bioengineering, School of Life Sciences, École Polytechnique Fédérale de Lausanne - EPFL, Lausanne, Switzerland; 7 School of Plant Sciences and Food Security, Tel-Aviv University, Tel Aviv, Israel

**Keywords:** Biophysical chemistry, Fluorescence resonance energy transfer, Protein aggregation

Correction to: *Nature Chemical Biology* 10.1038/s41589-022-01162-9. Published online 20 October 2022.

In the version of this article originally published, the Acknowledgements omitted thanks to the Swiss National Fund for grant number 200020_178763. The error has been corrected in the HTML and PDF versions of the article.

