# Supplementary material for: A fluorescent multi-domain protein reveals the unfolding mechanism of Hsp70
Source: Nat Chem Biol. 2022 Oct 20;19(2):198–205. doi: 10.1038/s41589-022-01162-9 (PMC9889267; doi:10.1038/s41589-022-01162-9)
Supplement: Supplementary file 1 — Supplementary Figs 1 and 2, and Supplementary Table 1. [file 41589_2022_1162_MOESM1_ESM.pdf]

nature chemical biology

Article

https://doi.org/10.1038/s41589-022-01162-9

A fluorescent multi-domain protein reveals the unfolding mechanism of Hsp70

In the format provided by the authors and unedited

Supplementary Information for:

A fluorescent multi-domain protein reveals the unfolding mechanism of Hsp70

Satyam Tiwari[1,2], Bruno Fauvet[2], Salvatore Assenza[3,4,5], Paolo De Los Rios[2.6,*] and Pierre Goloubinoff[1,7,*]

[1]Department of Plant Molecular Biology, Faculty of Biology and Medicine, University of Lausanne, CH-1015 Lausanne, Switzerland.

[2]Institute of Physics, School of Basic Sciences, École Polytechnique Fédérale de Lausanne—EPFL, CH-1015 Lausanne, Switzerland.

[3]Departamento de Física Teórica de la Materia Condensada, Universidad Autónoma de Madrid, E-28049 Madrid, Spain.

[4]Condensed Matter Physics Center (IFIMAC), Universidad Autónoma de Madrid, E-28049 Madrid, Spain

[5]Instituto Nicolás Cabrera, Universidad Autónoma de Madrid, E-28049 Madrid, Spain

[6]Institute of Bioengineering, School of Life Sciences, École Polytechnique Fédérale de Lausanne—EPFL, CH-1015 Lausanne, Switzerland

[7]School of Plant Sciences and Food Security, Tel-Aviv University, Tel Aviv, Israel

[*]Corresponding authors: pierre.goloubinoff@unil.ch, paolo.delosrios@epfl.ch

Supplementary Figures:

MAHHHHHHGSGEQKLISEEDLGSGSGSGGHHRVDFKTIYRAKKAVKLPDYHFVDHRIE
ILNHDKDYNKVTVYESAVARNSTDGMDELYKGASGGMVSKGEETTMGVIKPDMKIKLK
MEGNVNGHAFVIEGEGEGKPYDGTNTINLEVKEGAPLPFSYDILTTAFAYGNRAFTKY
PDDIPNYFKQSFPEGYSWERTMTFEDKGIVKVKSDISMEEDSFIYEIHLKGENFPPNG
PVMQKKTTGWDASTERMYVRDGVLKGDVKHKLLLEGSGMEDAKNIKKGPAPFYPLEDG
TAGEQLHKAMKRYALVPGTIAFTDAHIEVNITYAEYFEMSVRLAEAMKRYGLNTNHRI
VVCSENSLQFFMPVLGALFIGVAVAPANDIYNERELLNSMNISQPTVVFVSKKGLQKI
LNVQKKLPIIQKIIIMDSKTDYQGFQSMYTFVTSHLPPGFNEYDFVPESFDRDKTIAL
IMNSSGSTGLPKGVALPHRTACVRFSHARDPIFGNQIIPDTAILSVVPFHHGFGMFTT
LGYLICGFRVVLMYRFEEELFLRSLQDYKIQSALLVPTLFSFFAKSTLIDKYDLSNLH
EIASGGAPLSKEVGEAVAKRFHLPGIRQGYGLTETTSAILITPEGDDKPGAVGKVVPF
FEAKVVDLDTGKTLGVNQRGELCVRGPMIMSGYVNNPEATNALIDKDGWLHSGDIAYW
DEDEHFFIVDRLKSLIKYKGYQVAPAELESILLQHPNIFDAGVAGLPDDDAGELPAAV
VVLEHGKTMTEKEIVDYVASQVTTAKKLRGGVVFVDEVPKGLTGKLDARKIREILIKA
KKGGKSKLSYEQDGLHAGSPAALERAAAMDGGVQLADHYQQNTPIGDGPVLLPDNHYL
SYQSALSKDPNEKRDHMVLLEFVTAAGITLGMDELYKGGSGGMVSKGEELFTGVVPIL
VELDGDVNGHKFSVSGEGEGDATYGKLTLKLICTTGKLPVPWPTLVTTLGYGLMCFAR
YPDHMKQHDFFKSAMPEGYVQERTIFFKDDGNYKTRAEVKFEGDTLVNRIELKGIDFK
EDGNILGHKLEYNYNSHNVYITADKQKNGIKANFKIRHNIEGTDILQKKLEELELDE

Supplementary Figure 1: Amino acid sequence of MLucV (mTFP1-Luciferase-Venus). Cyan: the donor- mTFP1, Yellow: the acceptor- Venus, magenta: Luciferase.

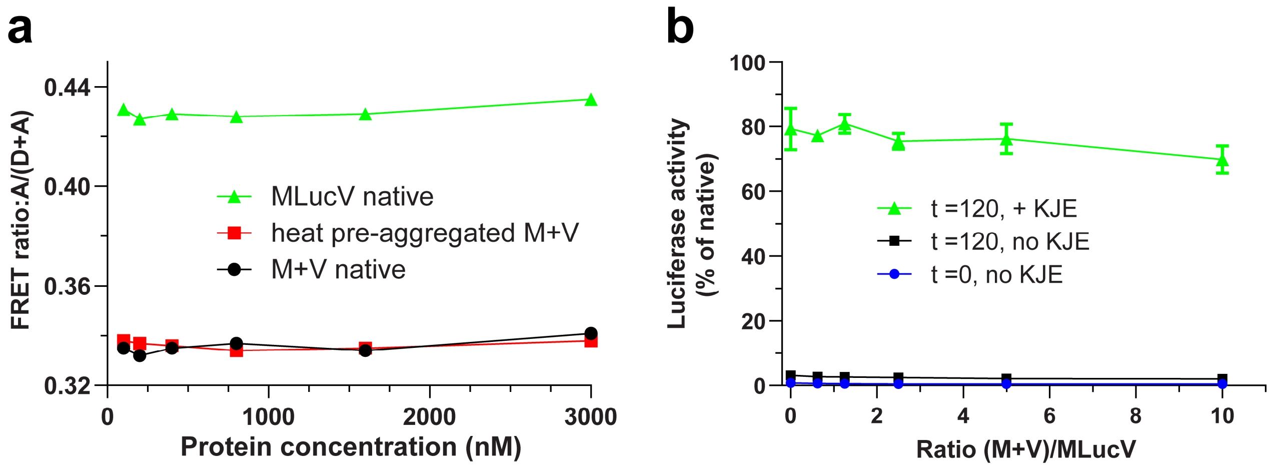

Supplementary Figure 2: The mTFP1 and Venus fluorophores do not show significant affinity for each other. **a)** FRET proximity ratios of increasing concentrations of native MLucV (green), 1:1 mixtures of separated fluorophores (M+V), under native conditions (black), or heat pre-aggregated (red). Heat pre-aggregated samples were prepared by incubation at 39°C during 12min followed by 20 min at 25℃. b) 5 μM MLucV in the presence of 0, 3.125, 6.25, 12.5, 25, or 50 μM equimolar mTFP and Venus were incubated in 4M urea at 25°C for 5min, then diluted 25-fold (to 0.2 μM MLucV) in buffer containing 4 μM BSA, 4 mM ATP and 4% glycine Betaine, without or with KJE (4:1:2 μM, respectively) and further incubated for 120 minutes at 25°C. Luciferase activity of controls without KJE was measured at t=0 and t=120 min; and the activity of samples with KJE(in green), was measured at t=120min. *These results imply that free native fluorophores have no affinity for the misfolded luciferases and for the native fluorophores of the MLucV aggregates.* Error bars in both panels show mean ± SD (n=3).
