# Supplementary material for: A fluorescent multi-domain protein reveals the unfolding mechanism of Hsp70
Source: Nat Chem Biol. 2022 Oct 20;19(2):198–205. doi: 10.1038/s41589-022-01162-9 (PMC9889267; doi:10.1038/s41589-022-01162-9)
Supplement: Supplementary file 2 — Reporting Summary [file 41589_2022_1162_MOESM2_ESM.pdf]

nature portfolio

Corresponding author(s): Pierre Goloubinoff
Paolo De Los Rios

Last updated by author(s): Aug 16, 2022

Reporting Summary

Nature Portfolio wishes to improve the reproducibility of the work that we publish. This form provides structure for consistency and transparency in reporting. For further information on Nature Portfolio policies, see our Editorial Policies and the Editorial Policy Checklist.

Statistics

For all statistical analyses, confirm that the following items are present in the figure legend, table legend, main text, or Methods section.

n/a	Confirmed	
☐	☒	The exact sample size (*n*) for each experimental group/condition, given as a discrete number and unit of measurement
☐	☒	A statement on whether measurements were taken from distinct samples or whether the same sample was measured repeatedly
☒	☐	The statistical test(s) used AND whether they are one- or two-sided<br>*Only common tests should be described solely by name; describe more complex techniques in the Methods section.*
☒	☐	A description of all covariates tested
☒	☐	A description of any assumptions or corrections, such as tests of normality and adjustment for multiple comparisons
☐	☒	A full description of the statistical parameters including central tendency (e.g. means) or other basic estimates (e.g. regression coefficient) AND variation (e.g. standard deviation) or associated estimates of uncertainty (e.g. confidence intervals)
☒	☐	For null hypothesis testing, the test statistic (e.g. F, t, r) with confidence intervals, effect sizes, degrees of freedom and P value noted<br>*Give P values as exact values whenever suitable.*
☒	☐	For Bayesian analysis, information on the choice of priors and Markov chain Monte Carlo settings
☒	☐	For hierarchical and complex designs, identification of the appropriate level for tests and full reporting of outcomes
☒	☐	Estimates of effect sizes (e.g. Cohen's *d*, Pearson's *r*), indicating how they were calculated

Our web collection on statistics for biologists contains articles on many of the points above.

Software and code

Policy information about availability of computer code

Data collection	Simulations were performed with LAMMPS (version 29Sep2021), patched with custom code implementing the potential for disordered proteins employed in the manuscript and available at https://github.com/saassenza/Hsp70Unfoldase/blob/master/IDP potential_LAMMPS_29Sep2021.zip. All ensemble FRET measurements were performed on a PerkinElmer LS55 fluorometer. Luciferase activity assays were performed on a Victor Light 1420 Luminescence Counter from Perkin-Elmer. SEC-RALS analysis were performed on an OMNISEC Resolve-Reveal system using OMNISEC 11.10 (Malvern Panalytical). Negative stain TEM Electron microscopy were performed on Philips CMl00 Biotwin (80kV) transmission electron microscope.
Data analysis	All PDB IDs of the models used to run molecular dynamics simulations are provided within the manuscript. Custom code was employed to extract distances between residues from the simulated trajectories. Graph Pad Prism 9, lmageJ 1.53k and Matlab R2019b were used to analyze data and to make figures. Code used to run and analyze molecular dynamics simulations, including sample LAMMPS inputs (including computation of distances on the fly) and the LAMMPS patch implementing the coarse-grained potential are openly available on GitHub at https://github.com/saassenza/Hsp70Unfoldase

For manuscripts utilizing custom algorithms or software that are central to the research but not yet described in published literature, software must be made available to editors and reviewers. We strongly encourage code deposition in a community repository (e.g. GitHub). See the Nature Portfolio guidelines for submitting code & software for further information.

Data

Policy information about availability of data

All manuscripts must include a data availability statement. This statement should provide the following information, where applicable:

- Accession codes, unique identifiers, or web links for publicly available datasets
- A description of any restrictions on data availability
- For clinical datasets or third party data, please ensure that the statement adheres to our policy

All data shown in the present study are available within the main and supplementary figures and provided as source data (Excel files) together with the manuscript. PDB IDs of the models used to run molecular dynamics simulations are provided within the manuscript. Maps and sequences of the plasmids generated in this study are available at https://doi.org/10.6084/m9.figshare.20502495. All generated plasmids and reagents are available upon reasonable request to the corresponding authors.

Human research participants

Policy information about studies involving human research participants and Sex and Gender in Research.

Reporting on sex and gender	Not applicable to our study
Population characteristics	Not applicable to our study
Recruitment	Not applicable to our study
Ethics oversight	Not applicable to our study

Note that full information on the approval of the study protocol must also be provided in the manuscript.

Field-specific reporting

Please select the one below that is the best fit for your research. If you are not sure, read the appropriate sections before making your selection.

☒ Life sciences ☐ Behavioural & social sciences ☐ Ecological, evolutionary & environmental sciences

For a reference copy of the document with all sections, see nature.com/documents/nr-reporting-summary-flat.pdf

Life sciences study design

All studies must disclose on these points even when the disclosure is negative.

Sample size	For all experiments, no sample size calculation was performed. All experiments were reproduced a minimum of n=3 times, which for our experiments sufficiently estimated the variability of the data.
Data exclusions	No data were excluded from our analyses
Replication	All experiments were fully reproducible and repeated at least three times. In particular, the reproducibility of the assays using heat-denatured and urea-preaggregated MLucV was ensured by repeating FRET and biochemical assays on different days, each time using a freshly-made preparation of MLucV aggregates, with reproducible results. Different protein preparations of MLucV were also used to see the reproducibility of the reporter construct.<br>For in-cell FRET assays in E.coli, at least six independent biological replicates were performed.<br>Electron microscopy experiments were reproduced at least three times, with consistent results.
Randomization	Randomization was not performed since it is not relevant to the types of experiments performed in our study (the states of the MLucV reporter protein were reproducibly different enough, as verified by independent experimenters in the lab, that any technical or subconscious biases could not account for the observed variations in the data)
Blinding	Blinding was not performed since it is not relevant to the types of experiments performed in our study, for the same reasons why randomization was not necessary.

Reporting for specific materials, systems and methods

We require information from authors about some types of materials, experimental systems and methods used in many studies. Here, indicate whether each material, system or method listed is relevant to your study. If you are not sure if a list item applies to your research, read the appropriate section before selecting a response.

Materials & experimental systems

n/a	Involved in the study	
☒	☐	Antibodies
☒	☐	Eukaryotic cell lines
☒	☐	Palaeontology and archaeology
☒	☐	Animals and other organisms
☒	☐	Clinical data
☒	☐	Dual use research of concern

Methods

n/a	Involved in the study	
☒	☐	ChIP-seq
☒	☐	Flow cytometry
☒	☐	MRI-based neuroimaging
