# Supplementary figures and images for: A fluorescent multi-domain protein reveals the unfolding mechanism of Hsp70
Source: Nat Chem Biol. 2022 Oct 20;19(2):198–205. doi: 10.1038/s41589-022-01162-9 (PMC9889267; doi:10.1038/s41589-022-01162-9)

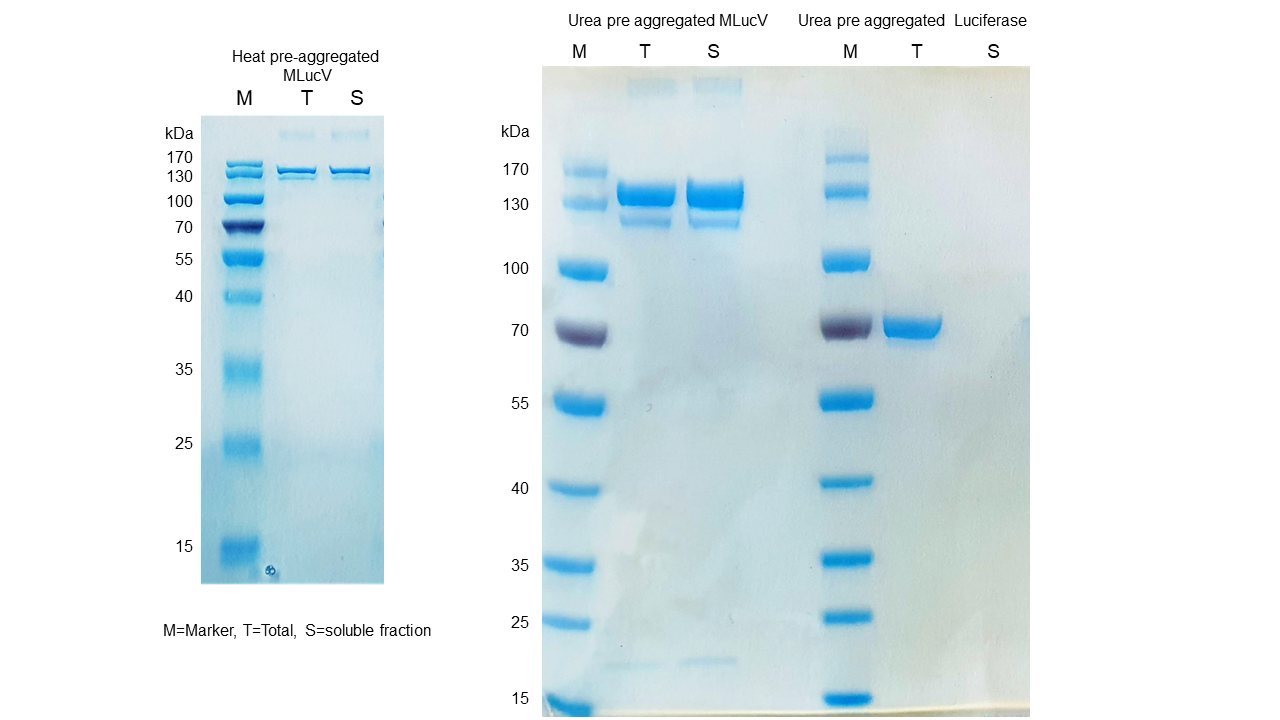

Supplement: Source Data Extended Data Fig. 2 — Unprocessed gel scans. [file 41589_2022_1162_MOESM10_ESM.tif]
